# A generic self-learning emotional framework for machines

**DOI:** 10.1038/s41598-024-72817-x

**Published:** 2024-10-28

**Authors:** Alberto Hernández-Marcos, Eduardo Ros

**Affiliations:** Research Centre for Information and Communications Technologies (CITIC-UGR) - Department of Computer Engineering, Automation, and Robotics (ICAR), University of Granada, Granada, 18071 Spain

**Keywords:** Emotions, Emotional model, Reinforcement learning, Emotional framework, Computational biology and bioinformatics, Neuroscience, Psychology, Mathematics and computing

## Abstract

In nature, intelligent living beings have developed emotions to modulate their behavior as a fundamental evolutionary advantage. However, researchers seeking to endow machines with this advantage lack a clear theory from cognitive neuroscience describing emotional elicitation from first principles, namely, from raw observations to specific affects. As a result, they often rely on case-specific solutions and arbitrary or hard-coded models that fail to generalize well to other agents and tasks. Here we propose that emotions correspond to distinct temporal patterns perceived in crucial values for living beings in their environment (like recent rewards, expected future rewards or anticipated world states) and introduce a fully self-learning emotional framework for Artificial Intelligence agents convincingly associating them with documented natural emotions. Applied in a case study, an artificial neural network trained on unlabeled agent’s experiences successfully learned and identified eight basic emotional patterns that are situationally coherent and reproduce natural emotional dynamics. Validation through an emotional attribution survey, where human observers rated their pleasure-arousal-dominance dimensions, showed high statistical agreement, distinguishability, and strong alignment with experimental psychology accounts. We believe that the framework’s generality and cross-disciplinary language defined, grounded on first principles from Reinforcement Learning, may lay the foundations for further research and applications, leading us toward emotional machines that think and act more like us.

**Supplementary Information:**

The online version contains supplementary material available at 10.1038/s41598-024-72817-x.

## Introduction

The capacity for emotions of varying complexity has evolved as an intrinsic and essential constituent of the mental processes in living beings displaying intelligent behaviors^1,2^. The reason is well supported by established disciplines such as neuroscience^3–5^, psychology^6–8^, and biology^1,9^: contrary to western philosophical tradition, which views them as detached from reason and hindering rational thought, emotions-and related phenomena like affects, feelings or sentiments-are currently understood as an evolutionary advantage, as first-order psychodynamic forces enhancing an organism’s adaptability to its environment, supporting the ultimate goal of survival. They play a crucial role in learning and in social behavior, and evidence suggests that damages in emotional components of the brain are severely detrimental to decision making^3^.

To date, though, these evolutionary advantages have not been effectively utilized in the field of Artificial Intelligence (AI), in which the study of emotions and feelings, vigorously developed since the 19th century, has had limited resonance. This stands in stark contrast to the profound influence exerted on AI (and vice versa) by breakthroughs from neuroscience, such as artificial neural networks^10,11^, bioinspired neural architectures^12,13^, and attention mechanisms^14,15^); biology, with examples like evolutionary algorithms^16,17^, multi-agent systems^18^ or swarm intelligence^19^; and psychology, for example goal-oriented behavior or reinforcement learning (RL)^20^. Instead, AI research has historically concentrated on emulating human reasoning, namely, the rational performance of our natural minds, relegating their emotional dimension to a second plane, or most frequently neglecting it. The field concentrated in the ascription of reason, or ‘cold logic’, to machines^21,22^.

But relevant voices have raised the issue that AI might be missing a key element to the flexibility, creativity, and efficiency of animal minds, suggesting a potential necessity for emotions in achieving true intelligence^23,24^. It has been argued that our current understanding of psychology and neuroscience brings into question the possibility of ‘pure rationality’ devoid of what we know as emotions^25^.

Early attempts to integrate emotions into AI focused on simulating their expression during human-computer interactions for more believable agents^26,27^, evolving into their detection in human inputs, such as emotion recognition from text, speech, or facial expressions^28–30^. Significant advancements have been made in this domain, particularly with the application of machine learning to physical and physiological signals like EEG (electroencephalogram), enabling more accurate cross-subject emotion recognition^31,32^. Additionally, the use of fuzzy logic or emotion-adaptive control systems in human-machine interfaces has expanded the capabilities of these systems, allowing for more nuanced interactions that account for emotional states^33,34^. With applications spanning brain-computer interfaces, empathic human-computer dialogue, assisted decision-making, and virtual reality^35^, the field is clearly making steady steps toward developing agents that can both understand and display emotions.

In contrast, much less progress has been registered in the complementary field of emotion synthesis, where this research is focused. Here, the fundamental challenge remains: creating AI agents that can elicit emotions truly comparable to natural ones. Most research in this area is predominantly theoretical^23,34,36–39^, with little practical advancement in learning, synthesizing, and integrating emotions in AI models and frameworks^25^. Even in RL, where some potential has been shown, the impact is limited, and current approaches often rely on pre-programmed, narrow emotion ranges or case-specific, arbitrary emotional models^40,41^. Innovative exceptions are rare and typically constrained to small, predefined emotion sets^42^, or tailored to specific environments, exhibiting limited generality^43,44^.

This slow integration of synthetic emotions in AI may have been caused by challenges in defining and measuring emotions, historical emphasis on rationalism over emotion^3^, and the success of classic, reason-based AI^45^. However, one major deterrent may be the absence of a universally accepted psychological framework explaining how emotions develop from raw inputs and prior learning, that specialists can adapt for AI^41^. Psychology itself lacks consensus on fundamental aspects, including the definition of emotions, their types, and terminology (emotion, affect, sentiment, etc.)^46^. Disagreements extend to the nature of emotions (discrete^6,47^, dimensional^7^, or mixed^48^), their triggers and physiological responses^8^, and whether they are universal^5,6^ or culturally dependent^49^.

The work presented here, building on key breakthroughs in these fields, aims to bridge this gap by defining such a framework from a computational perspective, motivated by the possibility that there lies an unrealized potential in truly emotional AIs.

## A generic self-learning emotional framework

### Inspirational background

Drawing on insights from neuroscience, psychology, and biology, we approach artificial emotions from a functional and information-theory perspective. We analyze the end-to-end dynamics that transform original perceptions into emotions, examining their triggers, intensity, sequence, and, critically for their synthesis, the cognitive abilities that each requires. Our goal is to reconcile, integrate, or extend existing AI frameworks and methods to understand and emulate emotions functionally, rather than replicating them at a low level.

Key factors that play a role in the emotional phenomena and guide our research include:

1. **Perception**: Rooted in neuroscience, perception is the initial stage of cognition, where sensory neurons capture external stimuli (like retinal photoreceptors or pain nociceptors). In combination with past experiences, perceptions are processed into increasingly abstract concepts, structuring a perceived reality into an internal representation^50^, crucial for emotional phenomena^51^.
2. **Reward and pain signals**: Emotions are linked to the limbic system, including structures like the hypothalamus and amygdala. The reward circuit and pain signals, forming the ‘common neural currency’^52^ guiding animal behavior^53^, are fundamental to emotional experiences. These subjective signals, our referential scale for what feels ‘good’ or ‘bad’, are shaped by individual homeostatic dynamics, maintaining the organism’s internal equilibrium^54,55^.
3. **Retrospection**: Past experiences heavily influence the emotional state, strongly correlated with recent outcomes^56,57^ and with the perceived sign of trend changes^58^. Additionally, repeated exposure to a reinforcing stimulus leads to ‘habituation’^59^, while neutral exposures result in ‘extinction’^60^.
4. **Anticipation**: Essential for their survival, animals predict future rewards using perception and memories, encoded in human neurons within the basal ganglia, midbrain, parietal, and cortex^61^, linked to dopamine-producing neurons^62^. Dopamine, key in reward prediction learning^63^, is widespread in animal phyla, with octopamine as its counterpart in Arthropoda^64^.
5. **Knowledgeability**: Some emotions may be associated with cognitive representations of the environment (such as surprise or curiosity)^65^, or with beneficial or harmful elements based on an individual’s subjective reality^66,67^.
6. **Feedback mechanism**: Lastly, emotions can act as signals, communicating internal states to the external environment and influencing interactions-such as fear or anger, signalling threats to others and prompting specific responses^1,6,68^. Internally, emotions can also guide behavior adjustments, maintaining balance and achieving goals^3,5^.
7. **Cognitive gradation**: All these factors engage specific brain regions and cognitive abilities, unique to each species, suggesting a progression or genealogy of emotions, as described in biology^1,69^.

### Introduction to the framework

In alignment with this foundational background, we propose here a generic, fully self-learning emotional framework for AIs that, based on first principles from the field of RL, allows any agent interacting with an environment to automatically learn, elicit and utilize its own synthetic emotional spectrum, convincingly resembling the natural emotions described in the literature. An overview of the framework is explained here, while its formal description and detailed methodology can be found in the supplementary section ‘Theoretical framework’. The framework is based on the following fundamental hypothesis:

**Hypothesis**: *All emotions correspond to distinct temporal patterns perceived in crucial values for a living being, such as recent rewards, expected future rewards or anticipated world states.*

Consequently, given that said crucial values, generated as cognitive variables, are determined by the individual’s specific cognitive abilities, they condition the complexity of the emotions experienced. The most basic ones reflect trends or patterns in reward / punishment signals, while increasingly sophisticated emotions integrate their subjectively anticipated values, anticipated world states, associations with other individuals or objects, etc. (Unlike in psychology or neuroscience, the term ‘reward’ encompasses positive and negative outcomes in RL, rarely using punishment for the latter, a convention that we also follow henceforth.)

This, in turn, suggests the viability of AI agents in RL automatically learning such patterns too, based on historical information-or subjective experiences-underpinning the self-learning framework introduced here.

#### Example

**Figure 1:**
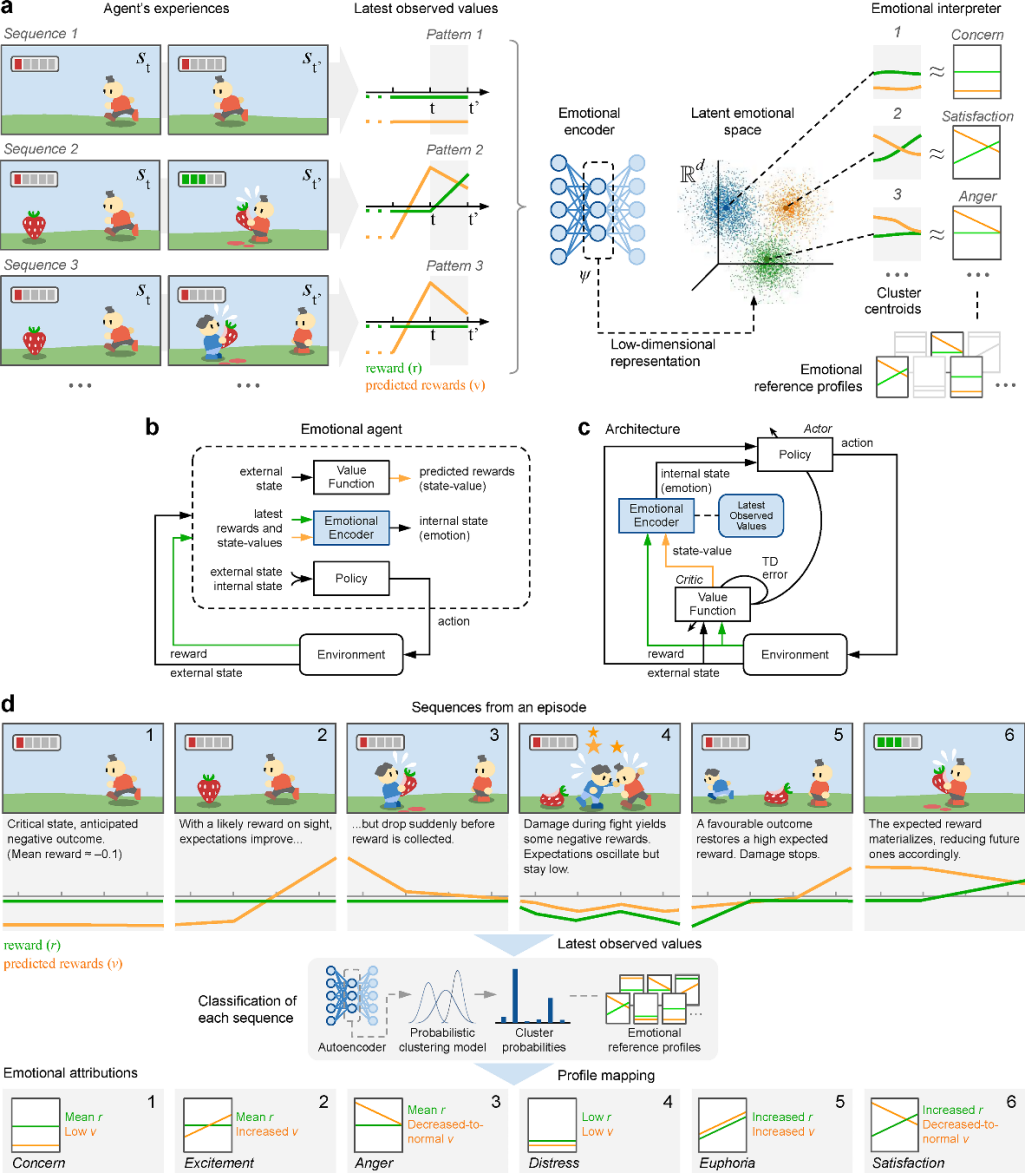
How emotions can be learned from experiences, then elicited and interpreted. (**a**), Learning: A reinforcement learning agent’s interactions are registered as sequences of multivariate time series (instantaneous *reward* and future predicted rewards-or *state-value*-in the example). An emotional encoder (such as a deep autoencoder) is trained unsupervised on the sequences, encoding them into a low-dimensional latent space. Distinct dynamic patterns in the sequences emerge as clusters whose centroids can be mapped to known emotional reference profiles (showing three here for simplicity). (**b, c**), Elicitation: The extended actor-critic architecture of an emotional agent integrates an emotional encoder storing the latest observed values (e.g., *rewards* and *state-values*), dynamically encoding ongoing sequences into instantaneous emotions. This encoded emotion extends the perceived state, enriching the policy’s input with an internal emotional state. Its training is not necessarily driven by the temporal difference (TD) errors. (**d**), Interpretation: Instantaneous emotions can also be mapped to known referential profiles for external interpretation or communication to other agents. A probabilistic clustering model can be used (e.g., Gaussian mixture) to predict their distribution probabilities over all clusters, associating them to the clusters’ preassigned profiles. The example interprets six consecutive profiles out of the 30 combinations in a *LOVE 2:5x6* interpretability mapping (two observed values with 5 and 6 possible patterns respectively; see supplementary ‘Interpretation of the learned emotions’ and Fig. 2 for details). (Figures created by Alberto Hernández Marcos.).

In Fig. 1 we introduce the main components of the proposed framework with an illustrative example of a simplified bioinspired RL setup. An AI agent’s goal is to survive in an environment where energy sources-the reward signal-are scarce and disputed with other agents. Its energy slowly diminishes over time (for example, average reward $\approx -0.1$), but it can perceive its nearby environment as a *state*, and is endowed with simple *actuators* for displacement, feeding and combat. Its cognitive abilities include a short-term-or *replay*-*memory*, a state-based prediction of future rewards-or *state-value function*-and a *policy* defining its behavior. As shown in Fig. 1a, the agent observes sequences of recent and predicted rewards during the simulation, producing differentiated temporal patterns as sequences of multivariate time series (MTS). We now describe how such patterns can be associated with emotions.

During a continuing successful execution without foreseeable hurdles, the agent would observe a series of positive rewards along with equally positive predictions, which might correspond to happiness. Contrarily, a disastrous and irreparable situation-such as a damaged actuator preventing progress-would yield a series of negative rewards and equally negative predictions, which might correspond to distress. Some other basic emotions might be equally described from similar stable patterns, or *emotional reference profiles*, like concern or optimism (top rows of Table 1).

**Table 1:** Association of some emotions to latest observed values.

Latest rewards	Latest predictions	Emotion	Rationale
Positive	Positive	*happiness*	Successful execution, no foreseeable hurdles
Negative	Negative	*distress*	Poor execution, no foreseeable improvements
Average	Negative	*concern*	Regular execution, expected to worsen
Average	Positive	*optimism*	Regular execution, expected to improve
...	...	...	...
Average	Increased	*excitement*	An opportunity emerged during regular execution
Decreased	Average	*frustration*	A reward stopped being received
...	...	...	...
Average	Decreased-to-average	*anger*	A threat arose, seen as potentially addressable
Average	Decreased-to-negative	*fear*	A threat arose, seen as hardly addressable

Crucially, within the framework, values are deemed *positive*, *average* or *negative* based on their comparison with historical observations, thus capturing the pivotal role of homeostasis in emotions (for instance, a positive reward that is substantially lower than the average reward would be considered as negative).

Other emotions can be associated with the dynamics of change, corresponding to recent trends of increases or decreases in these values, for example excitement and frustration (middle rows of Table 1). Finer interpretation of temporal trends allows the differentiation between closely linked emotions like anger and fear (bottom rows of Table 1), where cognitive appraisal of the agent’s control on its chances to overcome the challenge reflects a slight or steep drop respectively^70^.

We introduce the following additions to the classic RL setup (Fig. 1a):

- an *emotional encoder* (or emotional model) which, trained on various MTSs experienced, learns its latent features as a low-dimensional representation $\Psi _t\in \mathbb {R}^d$ representing the emotional state at time-step *t*;
- an *emotional interpreter* which, trained on the value distribution of $\psi$ over the latent space $\mathbb {R}^d$, maps its values to emotion terms for human interpretation, based on known reference profiles.

The use of the emotional encoder within an extended RL architecture allows the emotional agent to dynamically elicit instantaneous emotions, enriching the state used by an emotionally-enabled policy with a subjective, emotional state (Fig. 1b, c). For interpretability, the emotional interpreter can be used during the execution of the task to map instantaneous emotional states to known terms. Fig. 1d illustrates how a longer succession of events and their temporal patterns is associated with a series of coherent consecutive emotions. Richer emotional sequences in an actual RL environment, covering entire episodes, are discussed in ‘Results’.

**Figure 2:**
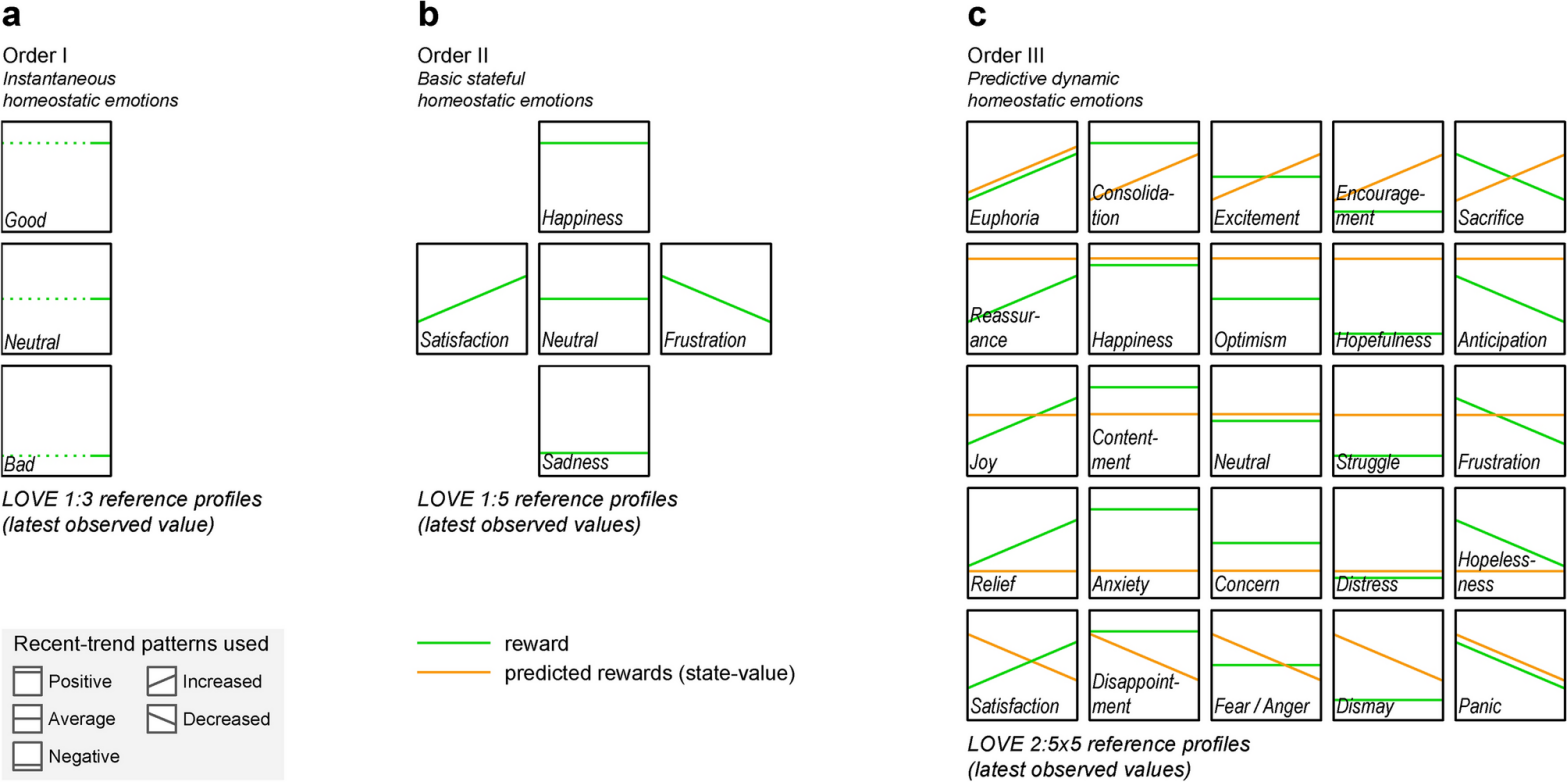
How cognitive abilities determine the emotional spectrum. A gradation of emotional spectra arises from increasingly higher cognition based on Latest Observed Values Encodings (or LOVE patterns) relative to subjective historical averages. (**a**), Order I. Immediacy: a single instantaneous *reward* value defines the simplest spectrum. (**b**), Order II. Retrospection: a short-term memory of the latest rewards allows elementary emotional dynamics. (**c**), Order III. Anticipation: the ability to predict future rewards (*state-value*) defines a much richer learnable spectrum of predictive dynamic emotions, which is used in the case study.

This illustrative example describes the core ideas of the framework based on a limited set of cognitive values, namely, reward and predicted rewards (or state-value). Agents endowed with higher cognitive abilities, like a world model anticipating future states, or social associations, would acquire more complex emotions, belonging to higher emotional orders, but are left out of this introduction (see overview in Fig. 2). A detailed definition of the framework can be found in ‘Theoretical framework’ (see Supplementary information), formalizing the concepts of emotion, emotional encoder, emotional spectrum, emotional orders (the agent in Fig. 1 was Order III), etc., as well as the detailed methodology applied to the case study.

In conclusion, we have introduced how the proposed framework captures the natural foundations described above, extending the RL framework to allow the learning, elicitation and utilization of synthetic emotions, as well as their external interpretation. The framework integrates objective perception (external state and rewards) with internal, subjective appraisals and the homeostatic definitions of *average* based on past experiences. The case study included in ‘Results’ illustrates how this synthetic emotional system naturally reproduces other well-known dynamics: elicitation/decay, coexistence, alternation, subjectivity, environment-dependency, and confusion.

## Results

### Application of the framework on a practical case study

The methodology was applied and tested in an RL case study using the classic *LunarLander-v2* environment^71^, selected for its simplicity (short episodes of 250–300 time-steps, and a maximum of 1000 for failed episodes), and the variety of life-or-death situations it presents for the simulated pilot, with potential for basic emotions spanning intense emotional ranges. Additionally, the lack of any emotional cues-no in-game character, face or body language is shown-guarantees an unbiased emotional attribution test.

**Figure 3:**
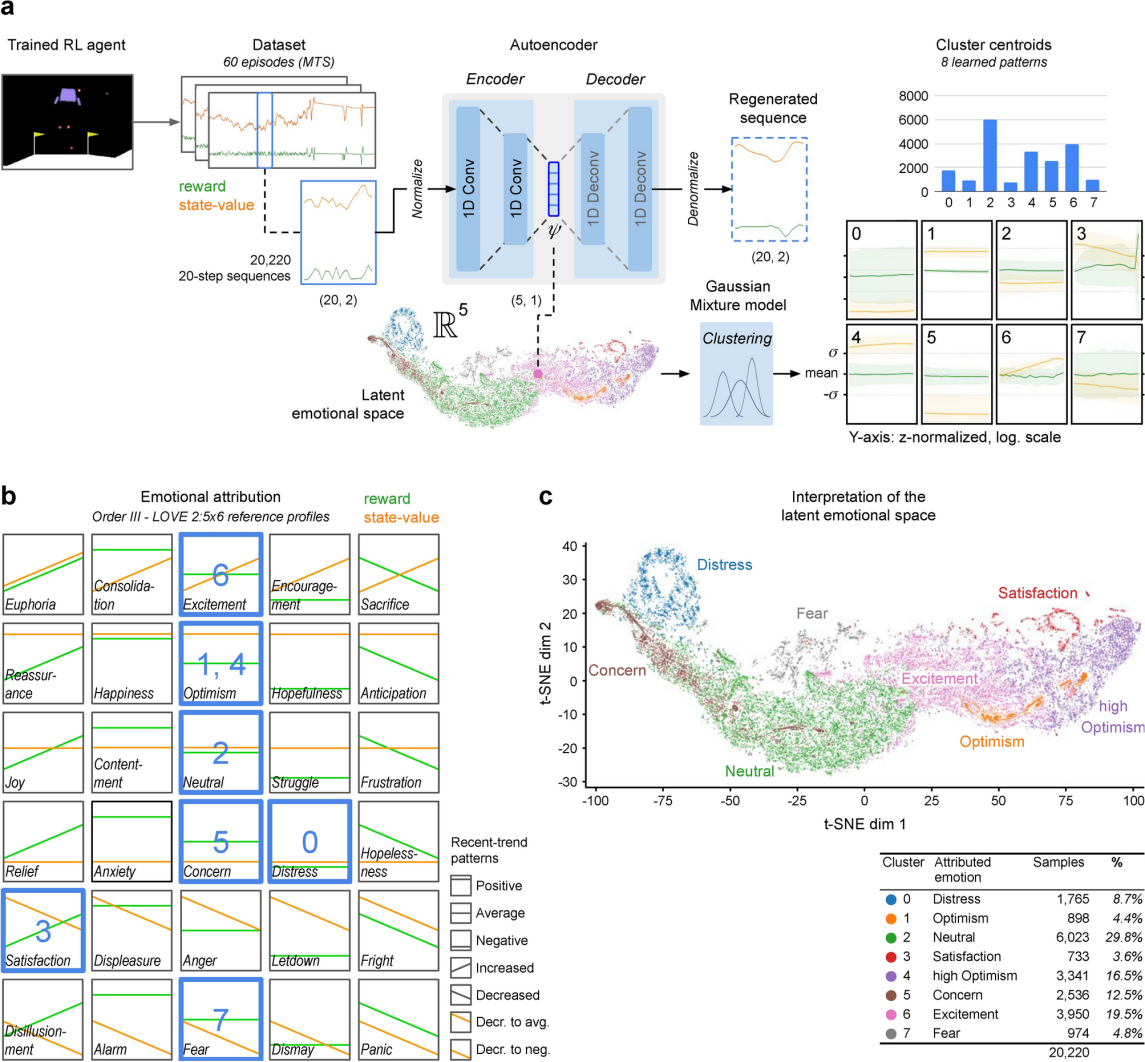
Learning emotions in a practical case study. The emotional framework was tested on the classic RL environment *LunarLander-V2*, on which an actor-critic PPO model (see “Methods”) had been previously trained to solve the task. (**a**), Learning the emotions from experience: The trained agent was run on new scenarios to obtain a dataset with stepwise values for *reward* and *state-value*. A 1D-Convolutional autoencoder was then trained on sequences from the dataset, and their low-dimensional latent representation obtained (as shown in the 2D graph using t-distributed stochastic neighbor embedding, or t-SNE). Finally, a clustering model identified eight distinct, uneven-sized clusters, whose prototypical sequences are shown on the right as 20-step average sequences of *reward* and *state-value*, where the shaded areas indicate the standard deviation. (**b**), Interpreting the learned patterns. Mapping the patterns against Order III - LOVE 2:5x6 reference profiles identified seven basic emotions, one of them in two degrees of intensity. (**c**), Interpretation of the learned emotional space. The distribution of the learned emotions over the latent emotional space is shown in this 2D t-SNE graph. The overlapping between the identified classes, originating from the continuous nature of the 5-dimensional values, is accentuated in their 2D representation.

By applying the steps detailed in ‘Methods’, the following two models were obtained (Fig. 3a):

- *Emotional encoder*: A deep autoencoder (DAE)^72^ was trained on 20,220 landing sequences experienced by a previously trained RL agent. The input values used were 20-step sequences of reward and state-value, in order to obtain an Order III emotional agent (see ‘Theoretical framework’ in the Supplementary information), generating their 5-dimensional step-wise representations.
- *Emotional interpreter*: A probabilistic Gaussian mixture model was trained on the 5-dimensional latent space learned by the emotional encoder, identifying eight distinct, uneven-sized clusters, whose centroids represented their respective prototypical multivariate sequences. For their interpretation, an Order III - LOVE 2:5x6 mapping was used (2 values, 5 reward $\times$ 6 state-value patterns, for more accurate mapping of clusters 3 and 7 than LOVE 2:5x5 in Fig. 2c).

**Figure 4:**
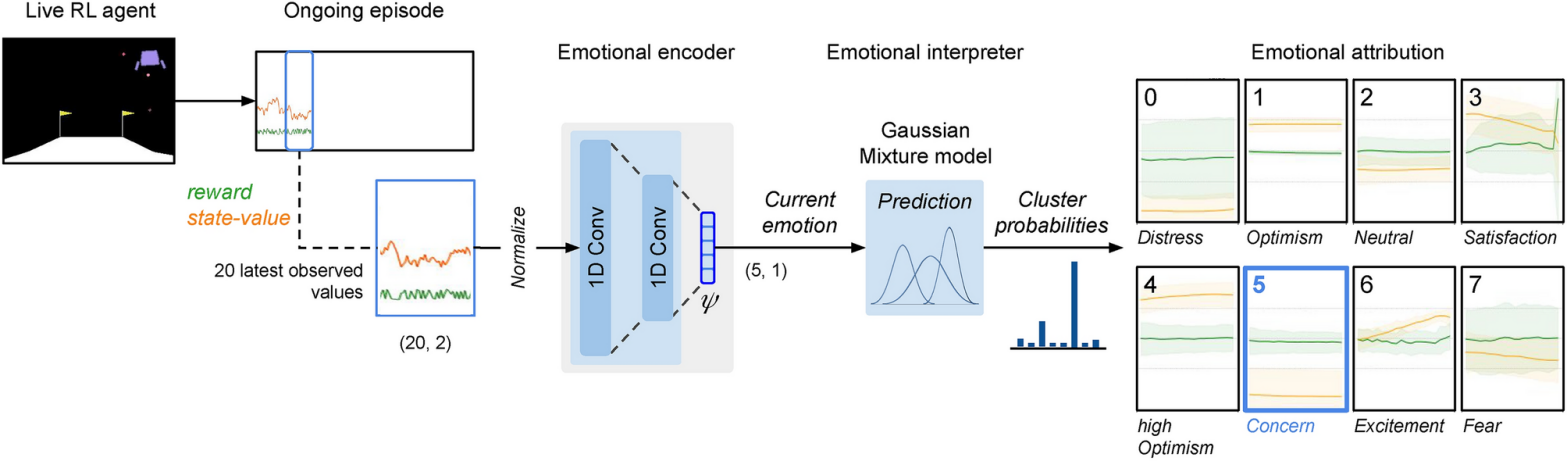
Interpretation of the instantaneous emotion. The emotional agent was tested on unseen scenarios and step-wise emotions synthesized by the learned emotional encoder based on the latest observed values of reward and state-value. The learned clustering model predicted the step-wise probability distribution of each encoded emotion over the eight clusters. In the image, where the agent is having difficulty aiming the spaceship toward the lunar base, a lower-than-average state-value (still unnormalized on the left) distinguishes ‘concern’ as the predominant emotion (cluster 5).

After the theoretical validation of its terms (see “Theoretical validation of LOVE profile terms”), the mapping was applied to the sequences, and seven differentiated basic emotions were identified (*distress, optimism, neutral, satisfaction, concern, excitement* and *fear*), one of them in two degrees of intensity (*optimism* and *high optimism*) (Fig. 4). The predominant class (cluster 2, with 29.8%), technically classified as neutral, shows a distinct below-average state-value and was treated as *neutral/slight concern*. As for the more clearly valenced emotions emerged, their distribution reflects its overall good competence at landing, with ‘positive’ emotions (optimism, satisfaction, high optimism, excitement) totalling a 44.1%, while ‘negative’ emotions (distress, concern, fear) only add up 26.1%.

The fully-interpreted emotional spectrum in Fig. 3c suggests clearly natural transitions, critical for the utility of the model, such as: (a) neutral $\rightarrow$ excitement $\rightarrow$ optimism $\rightarrow$ high optimism $\rightarrow$ satisfaction; (b) neutral $\rightarrow$ concern $\rightarrow$ distress; (c) neutral $\rightarrow$ fear, etc.

**Figure 5:**
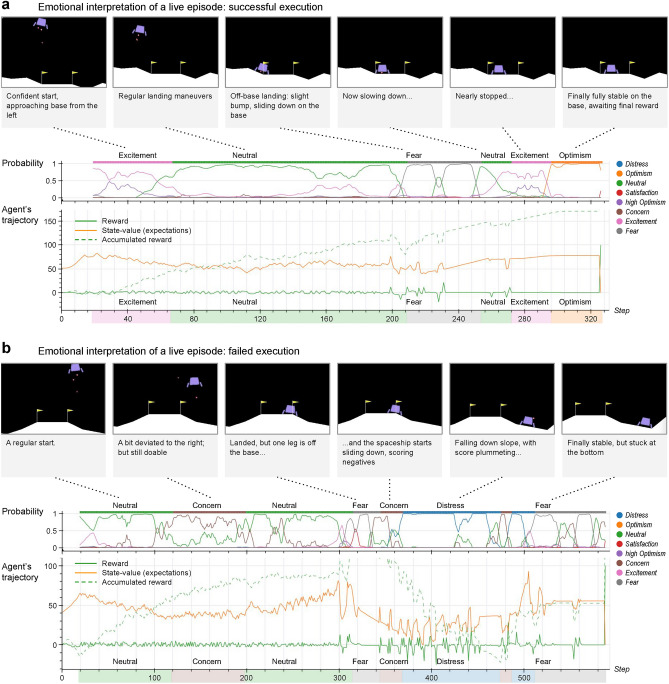
Interpreting live emotions. (**a**), Emotions during a successful landing. Emotional attributions start at step 20 (the time window of the encoder) and during this episode, positive emotions convincingly match the events experienced by the agent, who only faces a minor incident around step 200 (eliciting *fear*). Emotional transitions happen naturally as reward accumulates (with some hiccups in the 200–230 interval) and expectations vary, ending in the sequence *neutral-excitement-optimism*. The stepwise probabilities frequently allow predominant and secondary emotions to blend into richer states (like *excitement* and *high optimism* at the start). Notice how the smoothing applied imposes some latency to the attributions, but reduces instability (for example, a spurious *neutral* glitch at step 230). (**b**), Emotions during a failed landing. This episode starts with lower expectations than (**b**) (*excitement* fails to manifest itself), and *concern* is elicited at some point during the descent. Then the inaccurate landing in a precarious position produces *fear*, followed by *concern* and *distress* during the fall, as negative rewards accumulate down to a negative score. Once stabilized at the bottom, *fear* dominates the emotional state; notice though how the probabilistic attributions naturally elicit some sort of ‘emotional confusion’ in a hectic situation, with overlapping emotions.

To further validate its credibility, the agent was tested on unseen scenarios, step-wise emotions were synthesized by the emotional encoder and their probability distribution over the eight clusters was predicted by the clustering model (Fig. 4). Building on this, Fig. 5 vividly illustrates the stepwise elicitation and interpretation of the learned emotions during two complete episodes: a successful and a failed landing.

In summary, we found that the methodology spontaneously learned eight basic, recognizable emotions in an unsupervised manner. The synthetic emotional system naturally reproduced well-known natural emotional dynamics like:

- Elicitation and decay of step-wise emotions in synchrony with external changes observed and internal cognitive appraisals;
- Natural emotion transitions, with coexistence and progressive or sudden alternation driven by external and cognitive changes;
- Homeostasis, based on the agent’s subjective experience of average values registered;
- Subjectivity, with dependency on the individual’s appraisals (from the state-value function) and said homeostatic references.
- Environment dependency, with the emotional spectrum shaped and determined by the specific historical interactions between agent and environment;
- States of shock and confusion, with fast-overlapping negative emotions in highly unstable situations.

The results illustrate as well how, by associating instantaneous emotions with continuous values characterized by non-uniform distributions-which tend to give rise to clusters-the framework seamlessly integrates principles derived from both discrete and dimensional emotion theories, experimentally described in the literature^73^.

In this experiment, however, with very short-lived episodes, the documented dynamics of ‘habituation’ and ‘extinction’ were not reproduced, despite their feasibility within the framework, as analyzed in ‘Discussion’.

Finally, in most clustering models tried, two axes consistently aligned with key emotion dimensions from psychology: *pleasure *(or *valence*) and *arousal*. For instance, in Fig. 3c, the horizontal axis (pleasure) arranges emotions from negative (concern, distress, neutral/slight concern, fear) to positive ones (excitement, optimism, satisfaction, high optimism). The vertical axis (arousal) sorts emotions from low (neutral/slight concern) to high (distress, fear, satisfaction), with the others in-between. The significance of this alignment with historically documented emotion dimensions remains unanalyzed.

### Experimental validation of the learned emotions

#### Emotional attribution test with humans

To validate whether the synthetic emotions learned reflected true natural emotions, an emotional attribution survey was executed, comparing the subjective observations made by 96 independent participants during 48 different short sequences against their previously attributed emotion terms-concealed to them. The methodology used was Lang’s Self-Assessment Manikin (SAM)^74^, an extensively applied evaluation technique that directly measures emotional responses on three dimensions: *pleasure*, *arousal* and *dominance *(PAD)^75^, by rating each from 1 to 9 (see “Experimental validation of learned emotions with humans” for details and the Figure in ’Extended data’).

**Figure 6:**
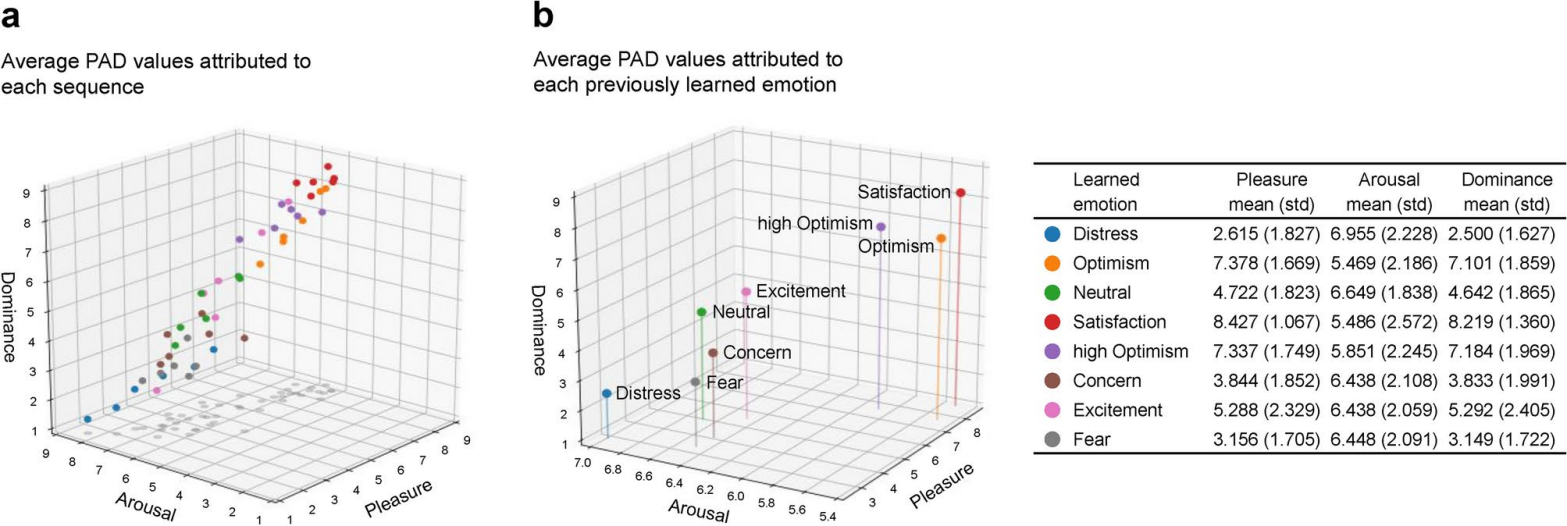
Results of the emotional attribution study. An emotional attribution survey ($n=96$) was conducted to rate the pilot’s emotion at the end of 48 short unlabeled sequences. For the test, Lang’s Self-Assessment Manikin (SAM)^74^ was used (see ‘Methods’), capturing the dimensions of *pleasure*, *arousal* and *dominance* (PAD) with Likert scales from 1 (lowest) to 9 (highest). (**a**), PAD attributed to each sequence. Unaware of the emotions previously attributed to the sequences, the participants consistently rated samples of the same class with similar PAD values, as illustrated by the color mapping. (**b**), Resulting PAD attribution to each emotion. The average PAD values of the videos associated with each emotion show a meaningful and coherent progression along the axes.

Upon analyzing the 2304 PAD data points registered, we observed that their average values, both by sequence (Fig. 6a) and attributed emotion (Fig. 6b), reflected a differentiated emotional spectrum. PAD values corresponded well with their associated emotions; for instance, sorting by pleasure shows a logical progression from negative to positive emotions: distress, fear, concern, neutral/slight concern, excitement, high optimism, optimism, satisfaction (with the exception of the two optimism states, similar in pleasure, distinguished though by the higher arousal attributed to high optimism). Notably, low arousal ratings were scarce, likely due to the dynamic, high-stakes nature of the sequences, with most ratings falling between 5 and 7.

The values for dominance, spanning from 2.5 to 7.2, were highly correlated with pleasure in this environment, and the ordering of emotions from ‘low’ to ‘high’ is almost the same: distress, fear, concern, neutral/slight concern, excitement, optimism, high optimism, satisfaction. The less clearly identified class was excitement, showing the highest dispersion in pleasure and dominance.

The test obtained a high reliability, with a high degree of agreement on PAD values for each video sequence across raters, according to the ICC2k statistical tests run (Intraclass Correlation Coefficient, two-way random effects model, absolute agreement) (see the Table in ‘Supplementary information’).

Equally remarkable, the underlying hypothetical emotions, despite being unknown to the raters, showed a high degree of statistical distinguishability according to the Hotelling’s T-squared pairwise tests run: optimism and high optimism were the least distinguishable emotions (with *p* = 0.116), followed by excitement and neutral (*p* = 0.002), while all other pairwise comparisons showed *p-*values well below 0.001 (see the Table in ‘Supplementary information’).

#### Mapping versus documented experimental accounts

Finally, to further validate the significance of the learned emotions, the PAD rates obtained for each emotion were mapped versus select pivotal experimental accounts from human subjects, numerically documented in psychology literature, obtaining significant agreement with some of the most broadly referenced PAD lists, selected by their mathematical qualities and impact in their field^75–79^.

To compare the PAD multivariate distribution obtained for each emotion with the referential PAD values, we applied statistical tests (Hotelling’s T-squared) and plain euclidean distance among means, focusing on the emotion terms of relevance for our context (short life-or-death landing maneuvers), excluding complex social, moral, self-conscious affects (such as kind, guilty, repentant), or the frequent non-emotion terms (like butter, cemetery, chair). We identified the three top matches in each account for each emotion, and then produced a *semantic collage* for each learned emotion with the five top matches across authors, obtaining the final mapping shown in Table 2:

**Table 2:** Top pleasure-arousal-dominance (PAD) matches across authors for each learned emotion.

Learned emotion	Top PAD matches across authors
Distress	Helpless, Scared, Nervous, Fearful(x2)
Optimism	Capable(x2), Optimism, Masterful, Skilled
Neutral/Slight Concern	Anxious, Startled(x2), Troubled, Intense
Satisfaction	Proud(x2), Confident, Safe, Achievement
High Optimism	Capable, Brave, Confident, Strong, Pride (feeling)
Concern	Confused, Nervous, Moody, Suspicious, Startle
Excitement	Aroused, Startled, Power, Anxious, Impulse
Fear	Insecure, Nervous, Thrill, Fearful, Fright

Remarkably, despite the disparity of terms, PAD values and applied mapping variants, significant agreement emerged between the originally attributed terms (from the LOVE 2:5x6 mapping) and their respective top matches across authors.

In summary, the videos previously associated with each emotion, invisible to the external raters, were described by them with consistent and differentiated values for their pleasure, arousal and dominance dimensions, validating their distinguishability and recognizability. The PAD values obtained could then be successfully associated with remarkably similar emotions described by their own PAD values in psychology literature.

## Discussion

In this work, we address emotions, a notably elusive concept across psychology, neuroscience, biology, and AI^35,80–82^. Our proposed framework’s novelty lies in its capacity to associate them with quantifiable, mathematically describable temporal patterns, akin to a ‘Periodic Table’ for emotions. This systematic categorization allows for an organized understanding and analysis of their spectrum, facilitating their integration into machine learning frameworks-a crucial step toward the goal of enabling artificial agents to generate and process their own emotions^83,84^.

Diverging from affective computing’s focus on external emotion recognition and communication to human subjects^28–30,35^-a rapidly evolving field thanks to the convergence of multiple technologies-our approach directly formalizes and integrates self-elicited synthetic emotions within practical emotional agents. Furthermore, unlike previous predominantly theoretical, case-specific, arbitrary or hard-coded models^23,34,36–44^, our transferable methodology learns them unsupervised within the RL framework, from first principles, and encompassing their entire spectrum, thus responding to a critical challenge identified in the field of AI^23–25,85^.

Crucially, the proposed framework is both analytically and experimentally verifiable, as shown in the ‘Results’. The emotional spectrum, learned during unlabelled agent interactions, was automatically grouped into eight basic emotions, such as distress, optimism, and satisfaction, obtaining strong empirical confirmation by subjective human observers. Participants, who were unaware of the emotions attributed by the system, rated pleasure-arousal-dominance (PAD) values during 48 sequences with high ICC2k agreement rates and, most relevantly, a strong alignment with the eight learned emotions (high statistical distinguishability in Hotelling’s T-squared tests), reflecting situational coherence as well as a natural progression from negative to positive emotions. PAD rates also showed significant semantic agreement with five key psychological studies in the literature.

The analysis of fully stepwise-interpreted episodes demonstrated that the learned emotions are also relatable to documented characteristics of natural emotions, such as the relevance of recent events, homeostasis-driven elicitation and decay, natural progressions aligned with external events and subjective appraisals, and the temporal coexistence resembling confusion in turbulent situations. The framework effectively integrates both discrete and continuous aspects of emotions by representing them as low-dimensional latent points, subsequently interpreted through probabilistic clustering. Additionally, the learned emotional spectrum is congruently shaped by the dynamics of the environment and the agent’s cognitive abilities. Future testing in new environments, yielding diverse emotional spectra, will further validate or challenge these findings.

The integration of emotional agents in RL, not explored in our initial experiments, shows promise. Incorporating learned synthetic emotions into the agent’s state, as detailed in ‘Extensions of the actor-critic method’, could increase its expected utility. Benefits similar to those in nature, like improved behavioral responses, efficient learning, and enhanced social competencies, are anticipated.

Currently though, the framework has only been tested on short-term ‘basic’ emotions, and has not been proved to capture retrospective dynamics like habituation (requiring more remote values) and extinction (requiring continuous learning of the agent’s functions), as well as emotional associations to external objects or subjects (like facilitators or blockers), knowledge-related emotions (like *surprise*) and higher-order emotions (such as social, moral or self-conscious).

‘Habituation’ simply requires the inclusion of moving averages as observed values-with continuous homeostatic renormalization-possibly combined with an expanded, smooth emotional window, while ‘extinction’ depends on the continuous training of the agent’s subjective state-value function on newly-neutral stimuli. More sophisticated cognitive abilities are required for the rest, as well as higher-order interpretability mappings and concurrent multiple-range windows, that are not discussed here.

Future testing might also target a broader coverage of the introduced mappings-not fully demonstrated in this first case study-or explore new patterns (like high/low decreases or policy dispersion degrees) and their potentially uneven prevalence.

More ambitious research might explore the extension of the framework to multiple reward setups, reflecting the heterogeneous rewards experienced by living beings (from physical sensations like *satiation* to higher-order feelings like *self-realization*). Likewise, tests incorporating partially observable environments, continuous learning, and multi-agent setups-exploiting social aspects of emotions-could enrich our understanding of the field and contribute to more comprehensive models.

Finally, in addressing ethical considerations, particularly whether machines should have emotions, our study clarifies that the emotions are synthetic and mathematically derived, with no AI experiencing actual suffering or enjoyment. However, the spontaneous emergence of emotions in AI, evidenced in apparently unrelated tasks-like next character prediction^86^-and suggested in RL as a byproduct of reward maximization^87^, cannot be ignored. Our framework provides interpretability tools for identifying and understanding such emergent emotions. Looking ahead, this clarity could be extended to computational models of empathy, predicting emotions of other agents or humans based on their approximate states and values.

We have introduced here a universal emotional language learned from first principles in reinforcement learning and inspired by primary cognitive variables like reward/punishment signals and predicted future values. The supporting theoretical framework and demonstrated methodology are, to the best of our knowledge, the first successful attempt to formally describe and synthesize a full range of recognizable, functional emotions of comparable characteristics to those of living beings. This pioneering work not only bridges the gap between artificial and natural emotional processes, but also opens new avenues for exploring the intersection of cognition, emotion, and machine learning. We believe that the generality of our framework could lay a foundation upon which further research and applications will lead us toward emotional machines that think and act more like us.

## Methods

### Application of the framework on a practical case study

Here we detail the exact step-by-step procedures followed to obtain the results described in ‘Results’ in the classic RL environment chosen, which can be applied to other RL setups with little modification.

#### Learning emotions from experience

**Pre-training of a conventional RL agent.** For simplicity, the *offline learning *approach was chosen, in which the emotional model is trained with the experiences collected by an already competent non-emotional agent. The open-source library *OpenAI’s Spinning Up*^88^, compatible with *OpenAI’s Gym*^71^, was chosen because its modular and well-documented implementation of RL algorithms facilitated the extensions required for the experiments.

The chosen method, actor-critic PPO (Proximal Policy Optimization)^89^, from the policy-gradient family, is broadly used for its stability during training, avoiding too large policy updates. Its training learns both a policy $\pi$ (the actor) and a value function *v* (the critic).

The non-emotional agent was trained to solve the task (average episode reward $\ge$ 200 over 100 consecutive episodes), with these features:

- Agent: Actor-critic PPO model, artificial neural network architecture: (64, 64), activation function: rectified linear unit (*ReLU*), seed: 10;
- Hyperparameters: *gamma*: 0.99, *lambda*: 0.97, *policy learning rate*: 0.0003, *state-value function learning rate*: 0.001, *target Kullback-Leibler (KL)*: 0.01.

The architectures explored involved combinations of 1-2-3 hidden layers, 32-64-128 neurons per layer, *ReLU*/*tanh* activation functions, and varied seeds.

**Dataset generation. Selection of input values and emotional window.** The trained agent was run on unseen scenarios to obtain a representative dataset of 60 episodes as MTS with stepwise values for a broad set of potential variables (reward, state-value, temporal difference, average reward, exponential moving average reward, and cumulative reward).

Upon review of the recorded episode dynamics and MTS, an Order III target mapping was chosen, for which only reward and state-value were required (see discussion of alternatives in the ‘Theoretical framework’, ‘Emotional orders’). (We anticipate that this mapping may perform well in a large variety of setups for its potential to capture a broad range of short-term, fundamental emotions with moderate, addressable complexity.) A tentative value for the emotional window was set at 20, expected to suffice to capture instantaneous emotions, and later corroborated by results.

The resulting dataset contained 20,220 20-step long sequences from the recorded MTS (training/test split = 16,281/3939). The two-variables (reward, state-value) were z-score normalized for training based on training-set statistics, thus establishing their average values as homeostatic references.

**Training of the emotional model from dataset sequences.** A 1D-Convolutional Autoencoder-a type of deep autoencoder (DAE)^72^ whose architecture is suitable for time series-was chosen for the task of representation learning (as explained in ‘Supplementary information’, ‘Model architecture’). The model was trained and tested on the dataset in an unsupervised manner to reproduce 20-step $\times$ 2 values normalized MTS sequences by learning their latent representation in a low-dimensional latent space^90^.

We used the *Keras/TensorFlow* library^91^, defining an encoder and a decoder with these features: *encoding_dim* = 5, *l1_filters* = 32, *l1_kernel_size* = 5, *l1_strides* = 2, *l2_filters* = 16, *l2_kernel_size* = 5, *l2_strides* = 2, *padding* = ‘same’, *activation* = ‘relu’. The separate encoder, used for emotion elicitation, consisted of 3,333 learned parameters. The training took 29 epochs with *batch_size* = 10 and *validation_split* = 0.1.

Various architectures and parameters were tested, including different encoding dimensions. Among these, an encoding dimension of 5 provided the best balance between reproduction root mean squared error (RMSE) and compression ratio. For this configuration, the RMSE values were 2.97119 for rewards and 4.32781 for state-values, with a compression ratio of 5:40. Although an encoding dimension of 10 resulted in lower RMSE values (2.48047 for rewards and 4.21228 for state-values), it produced a less favorable compression ratio of 10:40.

As noted, the model was not designed for input regeneration or denoising, but rather to capture high-level trends and magnitudes within the observed sequences. For context, the reward time series ranged from [− 100, 100], with a mean of 0.67 (standard deviation: 5.60), while the state-value time series spanned from [− 12.37, 109.51], with a mean of 62.41 (standard deviation: 18.99).

#### Elicitation of emotions

The trained emotional encoder obtained was used to encode the ongoing sequences of latest observed values from the full dataset, obtaining their 20,220 latent representations in the 5-dimensional latent emotional space. This was the emotional spectrum data used for interpretation. (The integration of the emotional encoder within the extended RL actor-critic architecture introduced in Fig. 1c was not addressed in this experiment.)

#### Interpretation of the learned emotions

**Clustering of the emotional spectrum.** To identify the distinct dynamic patterns in the emotional spectrum captured, a probabilistic Gaussian mixture model was trained on the latent space learned. This method models data as a mixture of a number of Gaussian distributions, capturing its covariance structure in clusters of uneven spatial extents, which suited the nature of the problem.

For the clustering of the latent space, we trained a probabilistic Gaussian mixture model from the *Scikit-learn* library^92^ and found the most promising clustering distributions to consist of 7 or 8 clusters, with minimal BIC scores (Bayes Information Criterion) and sufficient differentiation, although somewhat dependent on the initial random seed. The final choice was arbitrary, following practical experimentation on real sequences, and settled on 8 components with *covariance type* = ‘full’ (assigning to each component its own general covariance matrix). This approach exhibited satisfactory performance, thereby obviating the need for automation. The eight resulting classes, along with the average multivariate sequence representing their corresponding cluster centroids, are shown in Fig. 3a.

**Selection and validation of the interpretability mapping.** To map the eight resulting clusters with familiar emotion terms, the LOVE 2:5x5 mapping was initially tried (corresponding to the Order III emotional spectrum learned, with two values: reward and state-value (Fig. 2c), and then extended to LOVE 2:5x6 for a more accurate denomination of emotions 3 and 7 (Fig. 3b). This allowed the distinction between anger and fear based on the individual’s appraisal on its certainty and control on future outcomes (with anger associated with a decrease to average, and fear with a decrease to negative^70^).

The final terms for the eight emotions learned in this use case, as well as for the full set of thirty, were theoretically validated as described in ‘Theoretical validation of LOVE profile terms’, verified in live simulations and, finally, experimentally contrasted with external references (see ‘Experimental validation of the learned emotions with humans’).

**Attribution of emotion terms.** Based on the LOVE 2:5x6 mapping, the eight patterns were analytically associated with the following best-matching emotion terms (see Fig. 3a,b; note that the y-axis of the eight learned patterns is in logarithmic scale):

- Cluster 0: Distress (reward: below-average values; expectation: negative, well below $-\sigma$ values). The high variance of reward reflects very uneven values, which was deemed as subjectively negative due to the well-studied *loss aversion* principle (the pain of a loss is felt by individuals twice as intensively as the pleasure of an equivalent gain^93^).
- Cluster 1: Optimism (reward: average values; expectation: positive values around $+\sigma$).
- Cluster 2: Neutral/slight concern (reward: average values; expectation: below-average values). The most frequent emotion in this always uncertain environment (29.8% of the samples) falls closer to neutral than to concern, but the low expectation pattern justifies the compound naming.
- Cluster 3: Satisfaction (reward: increased values; expectation: decreased-to-average values). The least frequent emotion, triggered upon reception of a significant reward, with expectations decreasing accordingly
- Cluster 4: High optimism (reward: average values; expectation: well above $+\sigma$ values). Technically, both 1 and 4 match Optimism, but expectations in 4 significantly exceed $+\sigma$.
- Cluster 5: Concern (reward: average values; expectation: negative, well below $-\sigma$ values).
- Cluster 6: Excitement (reward: average values; expectation: increased values).
- Cluster 7: Fear (reward: average values; expectation: decreased to negative values).

Finally, the stepwise probabilities predicted by the emotional encoder were smoothened for more stable cluster attributions and easier external interpretation: a moving average of 5 steps on values and a minimal probability of 0.9 as reclassification threshold (or, alternatively, a minimal number of consecutive attributions of 10).

**Visualization.** To visualize the learned emotional space in both 2D and 3D, we used t-SNE (T-distributed Stochastic Neighbor Embedding) (see Fig. 3c for 2D and Section ‘Data availability’ for a 3D animation). For the 2D representation, the *Scikit-learn* library^92^ was employed with the following parameters: *seed* = 90, *n_components* = 2, *perplexity* = 200, *init* = ’pca’, and *n_iter* = 2000.

Although not technically required by the methodology, the use of colors to differentiate the classes learned by the emotional encoder was instrumental in the final selection of the autoencoder.

### Theoretical validation of LOVE profile terms

The principles described by the theoretical framework provide an initial foundation to associate LOVE profiles (idealized patterns of the latest values) with the best possible emotion terms in human language (see ‘Interpretation of the learned emotions’). However, given the difficulty of the task, their coherence was validated and refined both theoretically and experimentally. For the former, a sequence-coherence test was run, following this methodology:

1. Attribute an initial term to each of the 30 profiles, based on said theoretical principles.
2. Run offline simulations of event-guided, plausible emotional sequences where each profile is a state:
  Start from a stable state (for example, neutral).
  Try different sequences involving positive/negative evolutions of rewards and state-value (or expectations), with brief explanatory narratives.
  Discard beyond-scope transitions (such as positive $\rightarrow$ increased, or negative $\rightarrow$ decreased).
  Avoid too many abrupt transitions (positive $\rightarrow$ negative, negative $\rightarrow$ positive).
  End in stable or already visited states.
3. Review the resulting term sequences and repeat step 2 till fully natural transitions are obtained in all cases, leaving no pattern unused (ideally a few times).

For step 2, the sequence-coherence test was iterated and refined over thirty-eight simulated emotional sequences with full profile coverage, such as:

Neutral $\rightarrow$ (*An opportunity arises...*) $\rightarrow$ Excitement $\rightarrow$ (*and seems to hold.*) $\rightarrow$ Optimism $\rightarrow$ (*Suddenly the opportunity vanishes...*) $\rightarrow$ Anger $\rightarrow$ (*and we are back to normal.*) $\rightarrow$ END.

For additional clarity, a flow chart illustrating some examples of simulated emotional sequences is included in ‘Extended data’ under this same title.

### Experimental validation of learned emotions with humans

The following methodology was used for the emotional attribution survey and its ensuing mapping to psychology literature references.

#### Emotional attribution test with humans

**Dataset.** A representative list of 3–6 s long sequences was automatically selected from the dataset in which one specific learned emotion clearly prevailed over the others (six for each of the eight learned emotions, totalling 48 sequences at different stages of the landing maneuver). This guaranteed equal representation of all eight emotions (which presented some difficulty in the case of 3, satisfaction, the least frequent emotion, often smoothened out at sequence end by the probability smoothing applied).

To reduce the effect of fatigue on raters, the test was randomly split in two evenly-distributed lists of 24 videos (A and B), the sequence order further randomized in two versions each, and raters assigned alternating versions (A1, A2, B1, B2, A1, etc.).

**The tests with Lang’s SAM manikin.** All participants were adult volunteers who were native Spanish speakers, recruited from diverse academic and professional backgrounds. A subset of 26 participants received university credits as acknowledgment for their involvement. The study was conducted online, in Spanish language, and after registering and giving legal consent, an introduction was displayed explaining the dynamics of the study, the information shown during the sequences, the scoring system and the mission of the agent trying to land on a lunar base, described as a life-or-death task.

Once familiarized with the agent’s task, Lang’s Self-Assessment Manikin (SAM)^74^, a test extensively applied in psychological experiments and market research, was introduced to the participants. They were allowed to try it on two practice videos, characterizing a successful and a failed landing, whose respective results were discarded.

Finally, the subjects proceeded to the test, and for each of the 24 videos, reproduced on a separate screen, they were asked to describe the emotions they would associate to the state of the pilot at the end of each sequence using SAM. The test had no time limit, the videos could be played as many times as desired and ratings could be reviewed before the final submission.

SAM is a pictorial assessment technique that directly measures emotional responses on three main dimensions: pleasure, arousal and dominance^75^, associated with a person’s affective reaction to a wide variety of stimuli, typically by rating each dimension from 1 to 9 on a Likert scale. Some SAM tests incorporate a collection of words positioned at the relevant end of each Semantic Differential scale to identify the anchors of each dimension to the subject^94^. These original terms^95^ were predominantly translated to Spanish from the version by Gurbindo^96^, supplemented with nuanced contributions derived from the French version by Detandt^97^. The final terminology and set-up used is shown in ‘Supplementary information’.

**Statistical significance.** The study engaged raters aged between 18 and 64 (*n* = 96), with a fairly even split of 53 males and 43 females. The majority of participants (89) held a University degree, reflecting a diverse pool of individuals with varied educational backgrounds for comprehensive statistical analysis.

**Test reliability.** In order to assess the reliability of the ratings, the ICC2k statistic was used (Intraclass Correlation Coefficient, two-way random effects model, absolute agreement). The pleasure and dominance dimensions obtained ‘excellent’ correlation rates according to the orientative criteria by (Koo, 2016)^98^ (greater than 0.90) and (Cicchetti, 1994)^99^ (greater than 0.75), while arousal achieved ‘good’ per one guideline (between 0.75 and 0.90) and ‘excellent’ per another (greater than 0.75) (see Table in ‘Extended data’).

**PAD values attributed to the 48 videos.** Each of the 48 sequences was assigned a pleasure, arousal and dominance (PAD) triad of values, obtained as an average of the registered values, as shown in Fig. 6a. Tables with the obtained values and Pearson correlation values are included in ‘Extended data’.

**PAD values attributed to the eight learned emotions.** Similarly, each of the eight emotions was assigned a PAD triad of values as an aggregation over all raters from its six corresponding videos. The results can be visualized in Fig. 6b, and their values and correlations in ‘Extended data’.

**Distinguishability of the learned emotions.** To accurately validate the distinguishability of the eight emotions from their assigned PAD values, the robust Hotelling’s T-squared statistical test was used, comparing the distribution of each pair of multivariate samples.

#### Mapping versus documented experimental accounts

The PAD values of each learned emotion, obtained from human ratings, were compared to select pivotal experimental findings in psychology literature. From a diverse array of studies and reports, priority was given to those offering well-documented values for the three referential dimensions, showcasing the highest significance and impact within their field. The references chosen, all of them detailing mean and standard deviation for all three dimensions, were:

1. Russell-Mehrabian (1977) [RM]^75^: 151 emotional states. The terms seem to have been selected by the authors.
2. Bradley-Lang (1999) [BL]^76^: Affective Norms for English Words (ANEW), including 1,034 terms. The list contains very heterogeneous terms (like *abduction*, *abortion*, *absurd*, *abundance*, etc.) along with actual emotions.
3. Redondo (2007) [RE]^77^: Spanish ANEW; 1,034 Spanish words corresponding to the original ANEW with newly obtained PAD values.
4. Landowska (2018) [LA]^78^: ANEW-MEHR; 112 words selected from the Russell & Mehrabian’s list with the PAD values from ANEW.
5. Scott (2019) [SC]^79^: Glasgow Norms, including 5553 words. The list contains very heterogeneous terms (like *abattoir*, *abbey*, *abbreviate*, *abdicate*, etc.) along with actual emotions.

The task required overcoming a number of difficulties; firstly, we found a high degree of discrepancy in the terms included, heterogeneity of the emotional scopes, abundance of non-emotion terms, and other arbitrary peculiarities.

- Arbitrariness: the terminologies chosen by the authors, far from conforming a shared, standard set of emotions, often seemed inconsistently artificious (such as *weary with responsibility*, *quietly indignant*, *proud and lonely*, *snobbish and lonely*, in RM), or nuanced (for instance, *angry* and *angry but detached*; *hostile* and *hostile but controlled* in RM). All these terms were kept, despite producing somewhat heterogeneous top-match lists.
- Heterogeneity of the emotional scopes: All authors seamlessly mingled complex affects (like social, moral, self-conscious) with primary, instantaneous or more basic emotions (like *fright*, *anxious*, *euphoria*). For the purpose of tagging this agent’s task (short life-or-death landing maneuvers), we did not map the emotions associated with social relationships, moral judgements or self-conscious reflections, along with a few overly redundant or vague ones and one case of bodily needs (including *guilty*, *kind*, *repentant*, *lonely*, *hungry* in RM and LA; *unfaithful*, *loyal*, *insolent*, *admired* in BL and RE; *dignity*, *paranoid*, *emotional* or *achievement* and *achieved*, *frightened* and *fright* in SC).
- Non-emotions: The scope of some references was not limited to emotion terms, including all sorts of concepts (such as *butter*, *cemetery*, chair in BL and RE; *abdominal*, *apple (fruit)* or *musketeers* in SC), which were not used for emotional interpretation.

The terminology for the three dimensions has also historically differed among authors (namely, pleasure/valence; arousal/activation; dominance/control); however, we found that the more traditional (pleasure-arousal-dominance) model was easier to articulate and comprehend for non-expert participants.

As for the methodologies followed by the authors to obtain the PAD values, they also differed in format and profile of the participants, which probably contributed to the variance found in the reported values. For example, the different values reported for ‘angry’ (within the range of [− 1, 1]) are as follows:

- RM: (− 0.510, 0.590, 0.250)
- BL, LA: (− 0.538, 0.543, 0.138)
- RE: (− 0.700, 0.403, − 0.290)
- SC: (− 0.652, 0.227, 0.105)

Finally, despite all authors reporting standard deviations and number of samples, the lack of covariance matrices limited the applicability of standard statistical tests to compare two distributions, like Hotelling T-squared. To address this, we applied three different methods to map the PAD distributions obtained from our test for each emotion (sample 1) against reported PAD mean and standard deviation values (sample 2), often with unequal results in each table:

- Method 1: Hotelling T-squared test, assuming three independent variables in sample 2 (diagonal covariance matrix).
- Method 2: Hotelling T-squared test, assuming sample 2 had the same covariance matrix as sample 1.
- Method 3: Euclidean distance, comparing only the means.

To obtain the final mapping of each learned emotion, drawing inspiration from the ensemble of models concept, we independently mapped it against each reference table, identifying its three top matches, and then we merged the five top matches across authors into a semantic collage (see details in ‘Extended data’).

## Electronic supplementary material

Below is the link to the electronic supplementary material.

Supplementary Information 1.

## Data Availability

The data used in the case study, as well as some additional graphic material, are available on GitHub at https://github.com/Alberto-Hache/love-emotional-framework.

## Code availability

The code used in the learning, elicitation and interpretation of emotions in the case study is available on GitHub at https://github.com/Alberto-Hache/love-emotional-framework.
