# Publisher Correction: A generic self-learning emotional framework for machines

**DOI:** 10.1038/s41598-024-84229-y

**Published:** 2025-01-16

**Authors:** Alberto Hernández-Marcos, Eduardo Ros

**Affiliations:** Research Centre for Information and Communications Technologies (CITIC-UGR) - Department of Computer Engineering, Automation, and Robotics (ICAR), University of Granada, 18071 Granada, Spain

Correction to: *Scientic reports* 10.1038/s41598-024-72817-x, published online 28 October 2024

The Supplementary Information 1 file published with this Article contained an error where the author’s information was missing, subsections 1.3, 1.4 and 1.5 were placed under the wrong section and section “2.2 Theoretical validation of LOVE profile terms” was accidentally deleted.

The original Supplementary Information 1 file is provided below.

This error has now been corrected in the Supplementary Information 1 file that accompanies the original Article.

## Supplementary Information

Supplementary Information.

