# Supplementary material for: A generic self-learning emotional framework for machines
Source: Sci Rep. 2024 Oct 28;14:25858. doi: 10.1038/s41598-024-72817-x (PMC11519482; doi:10.1038/s41598-024-72817-x)
Supplement: Supplementary file 1 — Supplementary Information 1. [file 41598_2024_72817_MOESM1_ESM.pdf]

A generic self-learning emotional framework for machines: Supplementary information

Alberto Hernández-Marcos[1*] and Eduardo Ros[1]

[1]Research Centre for Information and Communications Technologies (CITIC-UGR), University of Granada, Calle Periodista Rafael Gómez Montero 2, 18071 Granada, Spain.

*Corresponding author(s). E-mail(s): albertoh@correo.ugr.es;

1 Theoretical framework

We cover here the full theoretical formalization of the introduced framework, its integration within the RL framework, and a detailed description of the generic methodology.

1.1 Definitions

In the classic Reinforcement Learning setup, an agent learns how to maximize the amount of reward received through interactions with an environment over a sequence of discrete time-steps. The actions influence not just the immediate rewards, but also the subsequent states of the system, with an impact on future rewards. The environment is formulated as a Markov Decision Process, satisfying the *Markov property*, that is to say, its response at $t+1$ depends only on the state and action taken at t [1].

The dynamics of this setup yield a series over time with the form: $S_0, A_0, R_1, S_1, A_1, R_2, A_2 \dots$ where S_t is the state observed by the agent at time-step t, A_t is the action then taken, and R_{t+1} is the reward observed after the action. We'll follow the classic notation in which capital letters are used for random variables, whereas lower case letters are used for the values of random variables (such as s, a, r, etc.).

The action A_t is chosen by a policy π based on state S_t. The literature offers a broad variety of methods to train π, but here we'll focus on the actor-critic methods [2], a subset of policy-gradient methods. These methods simultaneously obtain a policy π (the actor) and a value function v (the critic), whose learning is based on the temporal difference error, an error mechanism that has been compared by neuroscience to dopamine's in animal learning [3]. In this family of methods, widely used in state-of-the-art applications, v yields state-values, an estimate of future rewards, whose sign and magnitude are key for emotional elicitation.

These are the elements taken from the framework, whose detailed formulations are thoroughly studied in the literature [1]:

$S_t \in \mathcal{S}$ is the state observed at time t, from the set of states $\mathcal{S}$;
$A_t \in A$ is the action taken at time t, from the set of actions $\mathcal{A}$;
$R_t \in \mathcal{R}$ (a subset of $\mathbb{R}$) is the positive or negative reward observed at time t;
$\pi(a \mid s) \in [0, 1]$ is the probability of choosing action a in state s;
$v_\pi(s) \in \mathbb{R}$ is the value of state s under policy π (or expected future rewards).

In addition to the above, the following necessary definitions and components are introduced, whose possible implementations are separately addressed in 'Methodology':

Emotions. *Def.: The instantaneous latent representations, or encodings, of temporal patterns in vital values recently observed by the agent.* Said patterns can reflect significant trends in current, past and predicted values, endowing the agent with a meaningful, on-going appraisal on their progression. For

simplicity, the forthcoming exposition will focus on reward and state-value, briefly discussing promising alternatives further down.

Analogous to S_t (the external state observed at time t) we'll use the following notation:

$\Psi_t \in \mathcal{E}$ is the emotional state at time-step t, from the set of emotional states $\mathcal{E}$.

Emotional encoder. *Def.: A model that takes as input the multivariate time series sequence formed by the latest values observed by the agent, generating a low-dimensional encoded representation of their recent dynamics, namely, the above-mentioned emotion.* Its input would take the form of a multivariate time-series:

$$O_{t-T+1:t} = \{O^1, O^2, \ldots, O^W\}_{t-T+1:t}$$

where:

W is the number of observed values;
T is the length of the sequence, an arbitrary value that defines the emotional window of the agent;
$O^w_{t-T+1:t}$ is the time series sequence $(w_{t-T+1}, \ldots, w_t)$ of the latest T values observed for value w at time-step t.

In the sample case with two values, the sequence takes this form:

$$O_{t-T+1:t} = \{(R_i), (V_i)\}_{t-T+1:t}$$

where:

R_i and V_i are, respectively, the reward and state-value observed at time-step i within the latest T values at time-step t;

and the emotional encoder is formulated like this:

$$\Psi_t = e\left(\{(R_{t-T+1}, \ldots, R_t), (V_{t-T+1}, \ldots, V_t)\}\right)$$

where:

e is the emotional encoder (for example, a deep autoencoder);
$\Psi_t \in \mathcal{E}$ is a point in the latent emotional space $\mathbb{R}^d$ of dimension d, from the set of emotional states $\mathcal{E} \subset \mathbb{R}^d$, representing the emotional state at time-step t;
d is the dimension of the latent space learned by e, an arbitrary value or hyperparameter of the model.

Emotional window. *Def.: The lapse before which no observation has an emotional impact, imposed by the length of the input sequences processed by the emotional encoder, taken from the agent's experience.* Its choice is arbitrary, but too low or too high values might render the events within the time window unstable or irrelevant respectively. As a guidance, the analysis of the most basic natural emotions typically considers seconds or even minutes, rather than hours, just enough to keep relevant events within the short-term memory [4][5].

Emotional spectrum. *Def.: The range of possible values that the emotional state Ψ_t can take within the latent space $\mathbb{R}^d$, denoted as $\mathcal{E} \subset \mathbb{R}^d$.* It depends on: (1) the experiences from which it has been learned, namely, the environment and the actions taken in it; (2) the choice of values integrated in Ψ_t, limited by the agent's cognitive abilities, or emotional order. Despite taking continuous values, the distribution of the random variable over the latent space naturally may tend to follow non-uniform probabilistic distributions, amenable to interpretation.

Emotional orders. *Def.: A stratification of learnable emotional spectra of increasing cognitive complexity, determined by the set of values integrated in Ψ_t, which is limited by the agent's cognitive abilities.* The first, more basic orders, describe the gradation from the fleeting, irreflexive experience of instantaneous reward to multifaceted blends of expectations and recent events originated in the value function, replay memory and world model (see also Orders I, II and III in 'Introduction to the framework'):

Order 0: A standard RL agent; cognition allows action selection (by the policy π) based on sensory information (the state s). No emotions are elicited.

Order I - Immediacy: Taking *only the current value* of the reward defines the simplest learnable spectrum, with instantaneous homeostatic emotions such as good, bad or neutral (for positive, negative and average values respectively).

Order II - Retrospection: A simple short-term memory of the latest rewards endows the agent with elementary emotional dynamics, extending the learnable spectrum with basic stateful homeostatic emotions reflecting the latest trends (for example, satisfaction, frustration, happiness, sadness, or neutral).

Order III - Anticipation: The inclusion of the recent state- or action-values predicted by a value function can signal meaningful trends in future expectations, defining a much richer learnable spectrum of predictive dynamic homeostatic emotions (including anger, fear, concern, excitement, frustration, euphoria, etc.). RL algorithms that learn a value function are suitable here, such as SARSA, Q-Learning, actor-critic, etc.; we will focus on the latter in this study.

Order IV - World-knowledgeability: Agents that learn a world model (not necessarily for planning) can predict likeliest state continuations, detecting mismatches through state-prediction errors that can elicit basic knowledge-related emotions (such as surprise, curiosity, boredom), as well as valenced emotions towards objects or places (including aversion, attraction, phobia or fondness).

Higher Orders - Agents endowed with higher cognitive abilities, like longer-term memory or more complex world- or self-models, could learn and experience higher-order emotional spectra, like remote retrospections, social, moral or self-conscious emotions, which lay beyond the scope of this work.

A detailed analysis of the specific learnable emotions for each order requires the introduction of recent-trend patterns, discussed in 1.2.3, 'Interpretation of the learned emotions'.

Emotional interpreter. *Def.: A system that takes as input an emotion encoded by an emotional encoder, mapping it to known emotion terms for human interpretation.* The input would be $\Psi_t \in \mathbb{R}^d$, representing the emotional state at time-step t, and the output the attributed emotion term (for instance, 'joy', 'frustration', 'relief'), similar to the examples in 'Introduction to the framework'.

1.2 Methodology

Here we present how the framework operates, detailing a generic methodology for the spontaneous encoding of the emotional spectrum in an RL environment from raw agent's experience which, based on the definitions introduced, provides the following functionality:

1. Learning emotions from experience;
2. Elicitation of emotions and integration within an RL architecture;
3. Interpretation of the learned emotions.

1.2.1 Learning emotions from experience

Training of an emotional encoder. The simplest approach to training an emotional model (for example, a deep autoencoder) from direct observations of the agent is *offline learning*, following these steps:

1. Train a conventional RL agent A to the desired performance, for instance, with an actor-critic method.
2. Run the trained agent A on new episodes in the environment, saving the stepwise trajectories as MTS of the key values (for example, reward and state-value).
3. Train a deep autoencoder (unsupervised) on normalized trajectory sequences of length T, representing the emotional window.

However, if a new agent A' were to be defined and subsequently trained making use of the extended states (external + internal states), it would inherit the emotional dynamics of A, despite now learning a different policy and value function, which might impact its utility. The alternative would be the *online learning* of the emotional model along with the original training of the agent A (for example during the training cycles over the replay buffer of previous experiences), which despite the added complexity, might yield higher-utility emotions. In the real case covered in Results we adopted the simplest offline learning.

Input values. The choice of input values for the encoder is arbitrary, but will determine its learnable emotional spectrum. A logical prioritization inspired by nature and psychology, and based on the principles of utility and availability, is suggested here, following the order-wise stratification introduced by the framework (see 'Emotional orders' in section 1.1):

1. *reward*, as the key RL first principle. The objective value to maximize, defining 'good' and 'bad' for an agent, and directly received from the environment (but also applicable to less classic approaches like intrinsically-motivated RL [6]).
2. *state-value*, a prevalent cognitive product contributing contextual estimates of 'how good' future expected rewards will be. Its anticipatory nature adds valuable subjective charge to emotions like optimism or fear.
3. *state-prediction error*, quantifying the discrepancies between a learned world model and the observed state transitions. It can define emotions driven by an agent's understanding of its environment like surprise, astonishment or interest.

Beyond these values, related to orders I to IV, other possibilities might be considered:

4. *average reward*, a running estimate of $r(\pi)$, the average reward per time-step of the policy, typically expressed by $\overline{R}_t$ at time t, could provide a good measure of life-long performance and a broader reference of 'normality' for valenced emotions relative to its subjective 'neutral' or homeostatic value.
5. *moving average reward*, the mean value of rewards observed only during the latest steps, provides a more recent, adaptive reference of 'normality', and could substantiate the emotional dynamics of habituation to valenced emotions relative to its dynamic value, modulated by the length and parameterization set for the averaged period.
6. Other descriptive statistics of all the values above might capture emotionally-relevant dynamics (for instance, a high fluctuation in recent rewards should probably prevail over other values, with a sense of confusion or uncertainty).
7. Finally, *temporal difference error*, or TD error, estimating instantaneous step-wise errors in the expected future rewards, is applied all over the RL literature. However, it is by definition a byproduct of reward and value estimates [1], and therefore redundant. Furthermore, whenever predictions match observations well, TD provides little to no information regardless of the success or failures of the agent.

Model architecture. A natural choice for the unsupervised learning model that encodes recent sequences into a low-dimensional latent space is a deep autoencoder (DAE) [7]. This type of artificial neural network is extensively applied to learn compact feature spaces, which have been empirically demonstrated to capture existing similarities and relations among the original samples [8]. While the generative properties of the more sophisticated approach, variational autoencoders [9] are not required for the core task, interesting sequence-based alternatives like Recurrent Neural Networks [10][11] (dispensing with the fixed-size time window) or state-of-the-art self-attention models [12][13] might be considered in future works.

For the case of a DAE, since the purpose of the model is not input regeneration or denoising, an excessively high encoding dimension d, or too high depth and complexity of the architecture, might harm its purpose to capture high-level trends and magnitudes of the observed values.

1.2.2 Elicitation of emotions and integration within an RL architecture

The figure in 'Introduction to the framework' illustrates how a trained emotional encoder can be used by an agent to dynamically enrich its policy's input with an emotional state. The following definitions are proposed:

Extended state. *Def.: The extension of the state S_t observed from the environment at time-step t, or external state, with the emotion Ψ_t elicited by the emotional encoder, or internal state, with the form $X_t = (S_t, \Psi_t) \in \mathcal{S} \times \mathcal{E}$.* The extended state X_t can produce a richer representation that blends the objective and subjective perception of the agent at each step.

Emotional agent. *Def.: An RL agent whose policy makes use of an extended state X_t.* Its policy π would take this form:

$$\pi(a \mid x) \in [0, 1] \qquad \text{is the probability of choosing action } a \text{ in extended state } x \in \mathcal{S} \times \mathcal{E}.$$

In 'Introduction to the framework' we propose an architecture for such an emotional agent, based on the actor-critic method, that utilizes the extended state. While it is not included in this work, we provide the pseudocode for its training in a continuing task for agents of Order III and IV in section 3, 'Extensions of the actor-critic'.

1.2.3 Interpretation of the learned emotions

Finally, we detail here the principles to create a generic emotional interpreter. While interpretability of the learned emotions is not imperative for their utilization by an emotional agent, it significantly contributes to their validation and analysis, and potentially facilitates the external communication of the instantaneous emotional state. A few new concepts and tools are required for this purpose.

Clustering of the emotional spectrum. The distinct dynamic patterns learned by the emotional encoder will translate into a non-uniform distribution of ψ over the latent space, emerging as clusters whose centroids represent their respective prototypical sequences. The number and diversity of these clusters depends on many factors, including the clustering method chosen, but once learned, each centroid's sequence can be mapped to known *emotional reference profiles* associated with prototypical emotions described in the psychology literature, allowing human interpretation, as shown in 'Introduction to the framework'.

Selection and validation of the interpretability mapping. The applicable interpretability mapping is determined by the emotional order of the agent, and will allow the interpretation of the instantaneous emotions. The following definitions are proposed:

Emotional reference profile. *Def.: A possible combination of recent-trend patterns of the observed values in the corresponding emotional order associated to some known emotion term.* For instance, in Order III, where values include rewards and state-values (or expectations for more clarity), the profile < average rewards, positive expectations > might correspond to optimism. Other examples might be:

Latest observed trends	Emotion term
<average rewards, negative expectations>	*concern*
<negative rewards, negative expectations>	*distress*
<increased rewards, positive expectations>	*reassurance*
etc.	

Interpretability mapping. *Def.: A set of emotional reference profiles formed by the different possible combinations of their recent-trend patterns, ideally encompassing an ample range of emotion terms.* The following naming convention is followed for mappings:

$$\text{LOVE } N_{values} : N_{profiles}$$

where LOVE stands for Latest Observed Values Encoding. For example, a LOVE 2:5x5 mapping would correspond to two values (like reward and state-value) mapped over 25 profiles (the combinations of five patterns per value).

In 'Introduction to the framework' we showed possible interpretability mappings for the first three emotional orders, where each possible profile is associated with an emotion based on the interpretation of its recent-trend patterns. The set of recent-trend patterns defined for observed values is arbitrary, but a few basic ones inspired in psychology render significant variety and representativeness to the mapping:

average : Latest observed values do not differ much from their *historical average*;
positive : Latest observed values are *higher than average*;
negative : Latest observed values are *lower than average*;
increased : Latest observed values reflect a *positive trend*;
decreased : Latest observed values reflect a *negative trend*.

The three first patterns reflect the well-known principle of subjective 'normality' versus 'exceptionality' associated with homeostasis [14][15]. As for the emotional effect of recent changes perceived, research shows how trends may be more relevant than actual magnitudes (for instance, a minor improvement can

bring happiness to the unwell, yet a minor ailment may frustrate the healthy [16][17]), a principle that is captured in the two last patterns.

However, since the encoding of the latest values yields continuous values, specific criteria must be established for classification based on their statistical nature, for example: positive or negative when the mean value falls out of the range $[-\sigma, \sigma]$ (where σ is the standard deviation of the value); increased or decreased when the slope of the linear regression of the sequence deviates from its average value in the observed distribution by some amount (like its own standard deviation).

Profiles can nonetheless be extended for more nuanced interpretations if needed; for example, in Order III, the differentiation of 'decreased' into two patterns (decreased-to-average, decreased-to-negative) for state-value produces 30 profiles (LOVE 2:5x6), and the relevant distinction between fear and anger (see the emotional attribution mapping in 'Results'). Further possible extensions are briefly mentioned in 'Discussion'.

Attribution of emotion terms. The resulting profiles of each mapping can then be associated with known prototypical emotion (or affect) terms through analysis. However, given the lack of a broad consensus in the number, definition and categorization of emotions or affects in the literature [18][19][20], the following principles, partially inspired in the most influential theories, were applied to denominate the mappings of Orders I to III showed in 'Introduction to the framework'. Further research will possibly contribute refinements to these mappings:

– Conformity: Initial analysis of the value trends in each profile (magnitude, valence, stable / dynamic) for comparison with emotion descriptions in the relevant literature (including authoritative emotion/affect models, dimensions, lists and stability / transience).
– Meaningfulness: Prioritization of the most broadly adopted terms in the field, deprioritizing the uncommon.
– Comprehensiveness: Maximization of the overall emotional spectrum's range of each mapping.
– Cognitive adequacy: Contextualization within the respective cognitive orders (from basic, simple concepts in emotional Order I, to finer, richer emotions in Order III). Homogenization of the emotional spectra, not mingling complex affects (such as social, moral or self-conscious) with primary, instantaneous emotions, solely elicited by the recent events.
– Theoretical sequential coherence: Offline simulations of event-guided emotional sequences and review till fully natural transitions were obtained in all cases and all profiles visited several times (see 'Theoretical validation of LOVE profile terms' for details).
– Experimental sequential coherence: Finally, adaptation of the emotion terms to match the nature of the environment-agent interactions in the actual case study through the review of real emotional sequences.

Example: The pattern tagged as satisfaction is characterized by an increase in rewards, along with its corresponding decrease in future expected rewards or state-value, as described for example in (Schultz, 2015) [21]: "*Value informations for choices need to be updated when reward conditions change. For example, food consumption increases the specific satiety for the consumed reward and thus decreases its subjective value while it is being consumed.*"

Real-time emotional interpretation. Once the learned clusters have been associated to profiles from the LOVE mapping chosen, the instantaneous emotion encoded can be dynamically classified by the clustering model used and accordingly interpreted through its preassigned profile. If a probabilistic clustering model was used (for example, a Gaussian mixture model), a probability distribution will be obtained which, on top of the likeliest emotion, will capture richer nuances (for instance, 70% neutral, 30% fear may be interpreted as *slight fear*).

The stepwise stability of these classifications may be impacted by the different noisy values it is based on (environment rewards, model predictions, encoding model), which can be addressed by different techniques (such as smoothing moving average, reclassification thresholds, etc.). (See 'Application of the framework on a practical case study' for real-time interpretations in a concrete use-case.)

2 Extended data

2.1 Experimental validation of learned emotions with humans

PAD values attributed to the 48 videos. See Table 1 for the PAD values, and Table 2 for the correlation found among the three dimensions rated.

Table 1 PAD rates for each video in range [1, 9] aggregated over all raters

Video	Emotion	Pleasure		Arousal		Dominance	
		(mean)	(stdev)	(mean)	(stdev)	(mean)	(stdev)
A01	0	1.711538	0.976921	7.442308	2.199942	1.903846	1.332249
A02	0	1.230769	0.703364	8.115385	2.210969	1.480769	1.350242
A03	0	3.384615	1.561219	5.942308	2.118207	3.173077	1.617565
A04	1	8.211538	1.242018	5.288462	2.483961	7.980769	1.378979
A05	1	6.923077	1.569890	5.596154	1.763015	6.692308	1.698194
A06	1	8.211538	1.242018	5.096154	2.491237	8.115385	1.338038
A07	2	3.480769	1.552864	6.750000	2.085195	3.673077	1.700302
A08	2	5.615385	1.457285	6.134615	1.940576	5.500000	1.650906
A09	2	4.038462	1.825329	7.038462	1.596093	4.057692	1.808623
A10	3	8.500000	1.019419	5.019231	2.532072	8.403846	1.256506
A11	3	8.519231	1.244445	5.076923	2.936225	8.269231	1.509647
A12	3	8.115385	1.423250	5.557692	2.476663	7.788462	1.718829
A13	4	7.538462	1.954999	5.826923	2.332390	7.423077	2.295316
A14	4	7.673077	1.279111	5.692308	2.305479	7.192308	1.633455
A15	4	7.750000	1.631492	4.846154	2.452567	7.480769	2.033932
A16	5	4.384615	1.816632	6.519231	2.033932	4.557692	1.984385
A17	5	3.961538	1.385662	5.884615	1.986759	4.134615	2.067762
A18	5	3.038462	1.759696	6.923077	2.094307	3.115385	1.652732
A19	6	4.634615	1.680673	6.230769	1.722007	4.423077	1.933666
A20	6	6.500000	1.995092	6.057692	2.127444	6.846154	1.944168
A21	6	5.057692	1.719707	7.038462	1.888682	4.942308	2.090252
A22	7	3.423077	2.190408	5.923077	2.416010	3.192308	1.950751
A23	7	3.942308	1.764725	6.692308	1.754976	3.807692	1.749380
A24	7	3.076923	1.412079	6.442308	2.145798	3.173077	1.801102
B01	0	2.363636	1.699397	7.318182	2.066035	2.363636	1.526371
B02	0	4.204545	2.063859	5.931818	1.945749	3.522727	1.591966
B03	0	3.068182	1.909556	6.863636	1.959959	2.727273	1.436463
B04	1	7.636364	1.348400	5.477273	2.337646	7.090909	1.877878
B05	1	5.863636	1.936082	5.590909	1.821298	6.045455	1.790862
B06	1	7.204545	1.439954	5.840909	2.090323	6.409091	2.127540
B07	2	5.272727	1.436463	7.318182	1.307807	4.818182	1.742395
B08	2	4.409091	1.980347	6.386364	2.037051	4.409091	1.920734
B09	2	5.704545	1.439954	6.272727	1.689415	5.522727	1.620919
B10	3	8.818182	0.581607	5.500000	2.782838	8.636364	0.942311
B11	3	8.363636	0.685087	5.681818	2.310469	8.181818	0.994701
B12	3	8.272727	0.996824	6.227273	2.208479	8.068182	1.387615
B13	4	7.000000	1.524986	6.000000	2.090955	6.886364	1.781245
B14	4	7.431818	1.969509	6.090909	2.055262	7.568182	1.822313
B15	4	6.454545	1.810236	6.863636	1.664833	6.454545	2.028340
B16	5	3.590909	1.834022	7.090909	1.582141	3.181818	1.768888
B17	5	3.886364	1.768141	7.409091	1.574775	3.772727	1.903041
B18	5	4.227273	2.270781	4.795455	2.247268	4.181818	2.191662
B19	6	5.431818	1.619614	6.795455	1.862476	5.318182	1.681261
B20	6	7.772727	1.411969	6.113636	2.071017	7.568182	1.453108
B21	6	2.272727	1.420181	6.386364	2.553756	2.590909	1.674962
B22	7	2.545455	1.454017	7.181818	1.781986	2.636364	1.495589
B23	7	2.772727	1.178561	6.681818	1.877315	2.954545	1.524292
B24	7	3.000000	1.656558	5.818182	2.244090	3.000000	1.540160

Table 2 Correlation among Pleasure / Arousal / Dominance across all the videos (Pearson Two-sided)

X	Y	r	CI95%	p-unc	BF10	Power
Pleasure	Arousal	-0.69329	[-0.82, -0.51]	4.72E-08	3.42E+05	0.999933
Pleasure	Dominance	0.99436	[0.99, 1.00]	1.76E-46	1.49E+42	1
Arousal	Dominance	-0.688291	[-0.81, -0.50]	6.43E-08	2.56E+05	0.999913

Test reliability. See Table 3.

Table 3 ICC2k Intraclass Correlation Coefficients obtained from the study

Dimension	Test	ICC	F	df1	df2	p-value	CI95%
Pleasure	A	0.991303	143.006427	23	1173	< 0.001	[0.99 1.00]
Pleasure	B	0.987127	95.329519	23	989	< 0.001	[0.98 0.99]
Arousal	A	0.869813	10.420163	23	1173	< 0.001	[0.79 0.93]
Arousal	B	0.809354	6.115296	23	989	< 0.001	[0.69 0.90]
Dominance	A	0.987786	108.758808	23	1173	< 0.001	[0.98 0.99]
Dominance	B	0.984978	83.461934	23	989	< 0.001	[0.97 0.99]

PAD values attributed to the eight learned emotions. See Table 4 and Table 5 for the PAD values, and Table 6 for the correlation found among the three dimensions rated.

Table 4 PAD rates for each emotion in range [1, 9] aggregated over all raters from its six corresponding videos

Emotion	Pleasure		Arousal		Dominance	
	(mean)	(stdev)	(mean)	(stdev)	(mean)	(stdev)
Distress	2.614583	1.827222	6.954861	2.227804	2.500000	1.627293
Optimism	7.378472	1.669303	5.468750	2.186209	7.100694	1.859227
Neutral / Slight Concern	4.722222	1.822983	6.649306	1.838023	4.642361	1.864918
Satisfaction	8.427083	1.066547	5.486111	2.571585	8.218750	1.360491
High Optimism	7.336806	1.749395	5.850694	2.245072	7.184028	1.969494
Concern	3.843750	1.851611	6.437500	2.107623	3.833333	1.991270
Excitement	5.288194	2.329241	6.437500	2.059122	5.291667	2.404627
Fear	3.156250	1.704645	6.447917	2.091312	3.149306	1.721538

Table 5 PAD rates for each emotion normalized to range [-1, 1] aggregated over all raters from its six corresponding videos

Emotion	Pleasure		Arousal		Dominance	
	(mean)	(stdev)	(mean)	(stdev)	(mean)	(stdev)
Distress	-0.596354	0.456806	0.488715	0.556951	-0.625000	0.406823
Optimism	0.594618	0.417326	0.117188	0.546552	0.525174	0.464807
Neutral / Slight Concern	-0.069444	0.455746	0.412326	0.459506	-0.089410	0.466230
Satisfaction	0.856771	0.266637	0.121528	0.642896	0.804688	0.340123
High Optimism	0.584201	0.437349	0.212674	0.561268	0.546007	0.492374
Concern	-0.289062	0.462903	0.359375	0.526906	-0.291667	0.497818
Excitement	0.072049	0.582310	0.359375	0.514780	0.072917	0.601157
Fear	-0.460938	0.426161	0.361979	0.522828	-0.462674	0.430384

Table 6 Correlation among Pleasure / Arousal / Dominance across the eight emotions (Pearson Two-sided)

X	Y	r	CI95%	p-unc	BF10	Power
Pleasure	Arousal	-0.922272	[-0.99, -0.62]	1.11E-03	3.37E+01	0.961207
Pleasure	Dominance	0.999416	[1.00, 1.00]	4.99E-10	6.82E+05	1
Arousal	Dominance	-0.917867	[-0.99, -0.60]	1.30E-03	3.01E+01	0.955431

Distinguishability of the learned emotions. See Table 7.

Table 7 p-value of the Hotelling's T-squared statistical test for all emotion pairs

	D	O	N	S	H-O	C	E	F
D	-	-	-	-	-	-	-	-
O	3.66E-143	-	-	-	-	-	-	-
N	8.03E-44	4.32E-65	-	-	-	-	-	-
S	9.06E-211	1.04E-17	1.91E-123	-	-	-	-	-
H-O	3.34E-135	**1.16E-01**	6.79E-58	8.63E-17	-	-	-	-
C	4.71E-18	1.83E-92	4.69E-08	2.45E-155	8.91E-86	-	-	-
E	1.14E-49	1.36E-33	2.13E-03	1.62E-71	1.23E-28	3.48E-15	-	-
F	4.46E-06	2.37E-127	1.30E-25	1.57E-197	1.29E-118	1.39E-05	4.34E-33	-

Abbrev.: (D)istress, (O)ptimism, (N)eutral / slight concern, (S)atisfaction, (H)igh (O)ptimism, (C)oncern, (E)xcitement, (F)ear.

Mapping versus documented experimental accounts. Table 8 shows the top three matches of each learned emotion, with the highest match in bold text. Table 9 shows a *semantic collage* for each learned emotion with the five top matches across authors. These were the five referential PAD values used:

1. Russell-Mehrabian (1977) [22]: In this pivotal study, 151 different terms denoting emotional states were rated according to the three PAD values.
2. Bradley-Lang (1999) [23]: Affective Norms for English Words (ANEW), a lexicon of 1,034 frequent words providing "emotional ratings for a large number of words in the English language".
3. Redondo (2007) [24]: Spanish ANEW, with 1,034 Spanish words corresponding to the original ANEW with newly obtained PAD values.
4. Landowska (2018) [25]: ANEW-MEHR, with the assignment of the preexisting ANEW PAD values to 112 Russel-Mehrabian's emotions (out of 151).
5. Scott (2019) [26]: Glasgow Norms, including 5,553 words and PAD values.

Table 8 Results from mapping PAD values from the survey to five documented experimental accounts

Learned Emotion	Mapping to Documented Emotion-PAD Pairs (Top 3 Matches)				
	Russell-Mehrabian (1977)	Bradley-Lang (1999)	Redondo (2007)	Landowska (2018)	Scott (2019)
Distress	**Helpless**<br>Fearful<br>Insecure	**Scared**<br>Panic<br>Embarrassed	**Nervous**<br>Lost<br>Insecure	**Fearful**<br>Pain<br>Terrified	**Fearful**<br>Frightened<br>Panic
Optimism	**Capable**<br>Concentrating<br>Proud	**Optimism**<br>Masterful<br>Inspired	**Capable**<br>Easy<br>Confident	**Masterful**<br>Strong<br>Powerful	**Skilled**<br>Mighty<br>Pride (feeling)
Neutral / slight Concern	**Anxious**<br>Tense<br>Startled	**Startled**<br>Overwhelmed<br>Anxious	**Troubled**<br>Moody<br>Ecstasy	**Startled**<br>Anxious<br>Suspicious	**Intense**<br>Impulse<br>Urgent
Satisfaction	**Proud**<br>Capable<br>Self-satisfied	**Confident**<br>Triumph<br>Victory	**Safe**<br>Capable<br>Satisfied	**Proud**<br>Joyful<br>Masterful	**Achievement**<br>Courage<br>Triumphant
high Optimism	**Capable**<br>Concentrating<br>Strong	**Brave**<br>Pride<br>Bold	**Confident**<br>Capable<br>Interest	**Strong**<br>Powerful<br>Inspired	**Pride (feeling)**<br>Might (strength)<br>Drive (motivation)
Concern	**Confused**<br>Tense<br>Pain	**Nervous**<br>Overwhelmed<br>Suspicious	**Moody**<br>Thrill<br>Troubled	**Suspicious**<br>Confused<br>Startled	**Startle**<br>Risky<br>Shock
Excitement	**Aroused**<br>Concentrating<br>Anxious	**Startled**<br>Alert<br>Anxious	**Power**<br>Pride<br>Activate	**Anxious**<br>Aggressive<br>Curious	**Impulse**<br>Intense<br>Alert
Fear	**Insecure**<br>Confused<br>Pain	**Nervous**<br>Panic<br>Scared	**Thrill**<br>Fearful<br>Suspicious	**Fearful**<br>Despairing<br>Frustrated	**Fright**<br>Scary<br>Startle

Table 9 Top PAD matches across authors for each learned emotion

**Learned Emotion**	**Top PAD Matches Across Authors**
Distress	Helpless, Scared, Nervous, Fearful(x2)
Optimism	Capable(x2), Optimism, Masterful, Skilled
Neutral / Slight Concern	Anxious, Startled(x2), Troubled, Intense
Satisfaction	Proud(x2), Confident, Safe, Achievement
High Optimism	Capable, Brave, Confident, Strong, Pride (feeling)
Concern	Confused, Nervous, Moody, Suspicious, Startle
Excitement	Aroused, Startled, Power, Anxious, Impulse
Fear	Insecure, Nervous, Thrill, Fearful, Fright

Spanish adaptation of Lang's Self-Assessment Manikin (SAM) used. Fig. 1 shows a real screenshot with the graphic layout of the survey, with one of the videos rated during the test. Videos were hosted on *YouTube*, and results collected with *Google Forms* during June 2023.

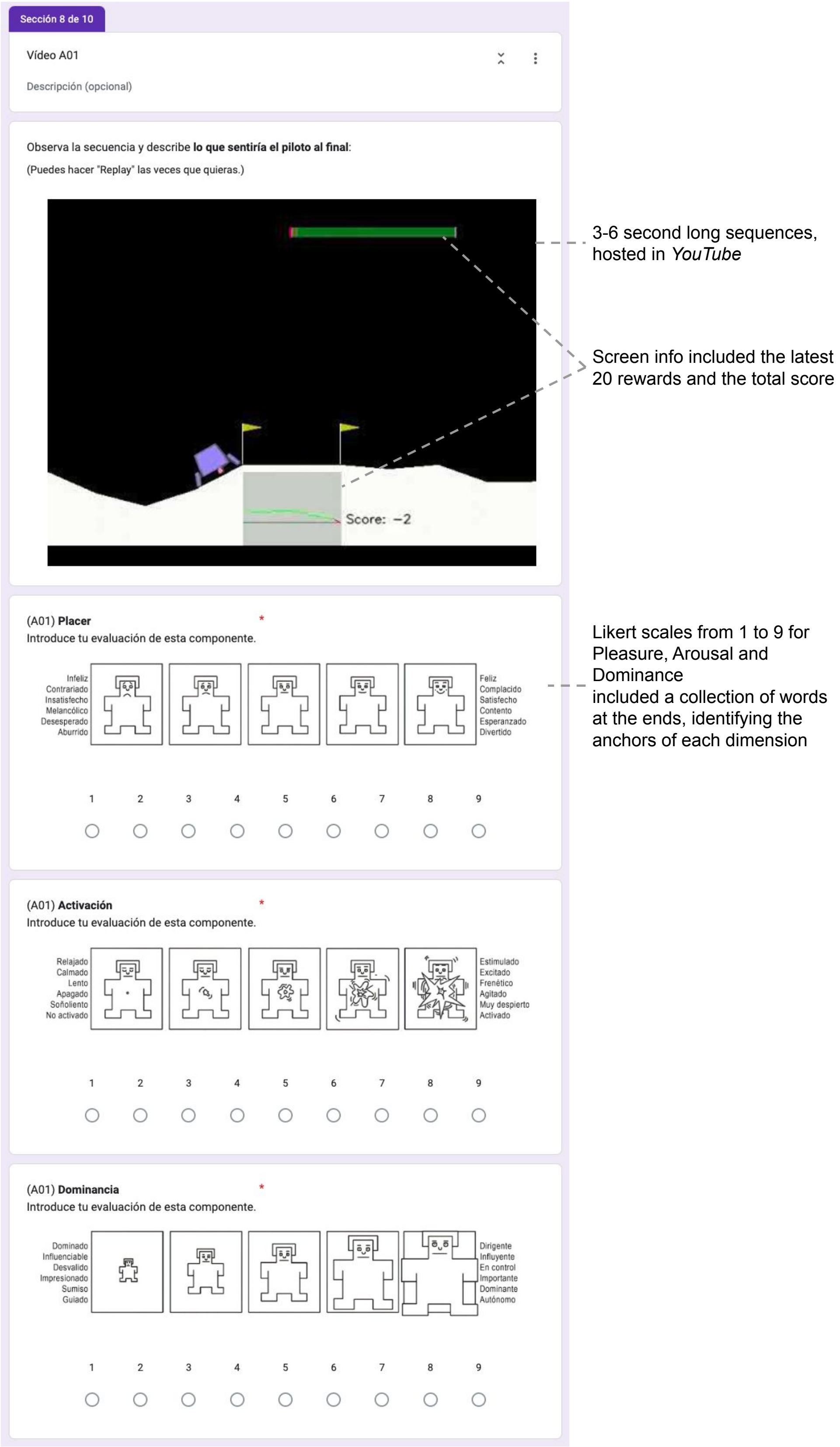

Fig. 1 **The graphic layout used for the rating of sequences.**

2.2 Theoretical validation of LOVE profile terms

Fig. 2 shows two of the 38 simulated emotional sequences used in the sequence coherence test for the validation of the LOVE 2:5x6 interpretability mapping.

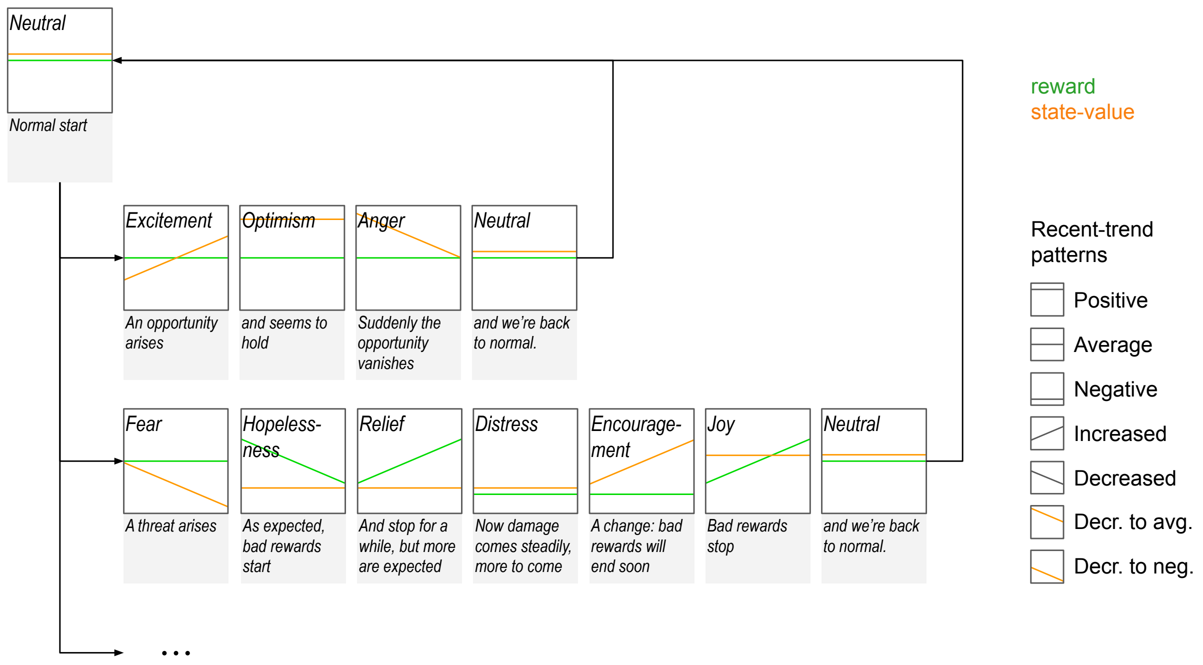

Fig. 2 Theoretical validation of LOVE profile terms. Each node represents an emotional reference profile from the LOVE 2:5x6 interpretability mapping. The simulation of all possible generic sequences, combining series of recent-trend patterns, helped to validate the congruence of the emotion terms attributed to each emotional reference profile.

3 Extensions of the actor-critic method

We document here the pseudocode for training Order III and Order IV emotional agents, based on the architecture introduced in 'Introduction to the framework' (the significance of Orders I and II is more theoretical than practical). We followed the notation and original pseudocode taken from (Sutton, 2018) [1]. For the sake of brevity, we focus on the continuing task setup—which will apply better to future biologically-inspired natural setups—but the extensions can be applied to all the variants, such as episodic tasks, eligibility traces, etc.

Training of a classic actor-critic agent (Order 0 - Non-emotional agent)
As a reference, we first include Algorithm 1, the original pseudocode for the training of the policy and the state-value function of a vanilla actor-critic agent for continuing tasks (based on the average reward estimates, and disregarding the classic *gamma* discount factor) [1]. This would correspond to a non-emotional agent (with emotional Order 0). In the pseudocode provided:

$S, S' \in \mathcal{S}$	are the states observed at time-steps t and $t+1$ from the set of external states $\mathcal{S}$;
$A \in \mathcal{A}$	is the action taken at time-step t, from the set of actions $\mathcal{A}$;
$R \in \mathbb{R}$	is the reward observed at time-step t;
$\overline{R} \in \mathbb{R}$	is the estimate of average reward at time-step t;
$\pi(a \mid s, \boldsymbol{\theta}) \in [0,1]$	is the probability of choosing action a in state s given parameter vector $\boldsymbol{\theta}$;
$\widehat{v}(s, \mathbf{w}) \in \mathbb{R}$	is the approximate value of state s given weight vector $\mathbf{w}$.

Algorithm 1 Training of a vanilla non-emotional actor-critic agent (continuing tasks)

Input: a differentiable policy parameterization $\pi(a \mid s, \boldsymbol{\theta})$
Input: a differentiable state-value function parameterization $\widehat{v}(s, \boldsymbol{w})$
Initialize $\bar{R} \in \mathbb{R}$ (e.g. to 0)
Initialize state-value weights $\boldsymbol{w} \in \mathbb{R}^{d'}$ and policy parameter $\boldsymbol{\theta} \in \mathbb{R}^{d''}$ (e.g., to $\mathbf{0}$)
Algorithm parameters: $\alpha^w > 0$, $\alpha^\theta > 0$, $\alpha^{\bar{R}} > 0$
Initialize $S \in \mathcal{S}$ (e.g. to s_0)
loop forever (for each time-step)
  $A \sim \pi(\cdot \mid S, \boldsymbol{\theta})$ ▷ Choose an action
  Take action A, observe S', R ▷ Observe new state and reward
  $\delta \leftarrow R - \bar{R} + \widehat{v}(S', \boldsymbol{w}) - \widehat{v}(S, \boldsymbol{w})$ ▷ Temporal difference error
  $\bar{R} \leftarrow \bar{R} + \alpha^{\bar{R}}\delta$ ▷ Update average reward
  $\boldsymbol{w} \leftarrow \boldsymbol{w} + \alpha^w \delta \nabla \widehat{v}(S, \boldsymbol{w})$ ▷ Adjust $\widehat{v}$
  $\boldsymbol{\theta} \leftarrow \boldsymbol{\theta} + \alpha^\theta \delta \nabla \ln \pi(A \mid S, \boldsymbol{\theta})$ ▷ Adjust π
  $S \leftarrow S'$ ▷ Update state
end loop

Note that, for greater clarity, the pseudocode does not optimize the number of invocations to the function $\widehat{v}$. This is also true for the forthcoming versions of the algorithm involving other functions.

Training of an emotional agent (Order III - Anticipation) An emotional agent of Order III would be endowed with an emotional encoder e, trained on reward and state-value MTSs, based on the extended architecture introduced in 'Introduction to the framework'. Algorithm 2 shows the pseudocode to train the agent, based on and emotional encoder e previously trained offline, where:

$R^{(T)} = [R_{t-T+1}, \ldots, R_t] \in \mathbb{R}^T$	is the list of the latest T rewards R observed at time-step t;
$V^{(T)} = [V_{t-T+1}, \ldots, V_t] \in \mathbb{R}^T$	is the list of the latest T state-values V observed at time-step t;
$\Psi = e(R^{(T)}, V^{(T)}) \in \mathbb{R}^d$	is the emotion encoded by e from $R^{(T)}$ and $V^{(T)}$ at time-step t;
$X = (S, \Psi)$	is the extended state observed at time-step t;

$\pi(a \mid x, \boldsymbol{\theta}) \in [0,1]$ is the probability of choosing action a in extended state x given parameter vector $\boldsymbol{\theta}$;

T is the emotional window of the agent.

The implementation of the short-term memory utilizes a circular buffer storing only the latest T observed values, which serve as the input to e. This is needed for a fixed-input architecture of e, but may not be required in other implementations, such as a Recurrent Neural Network, whose hidden state naturally represents past inputs. For the same reason, the first $T-1$ time-steps require special treatment until the two input lists have accumulated enough values. The simplest approach is to assign an arbitrary initial value ψ_0, for example, corresponding to a neutral emotion.

Algorithm 2 Training of an Order III emotional actor-critic agent (continuing tasks)

Input: a differentiable policy parameterization $\pi(a \mid s, \boldsymbol{\theta})$
Input: a differentiable state-value function parameterization $\widehat{v}(s, \boldsymbol{w})$
Input: a trained emotional encoder $e(R^{()}, V^{()})$
Initialize $\bar{R} \in \mathbb{R}$ (e.g. to 0)
Initialize state-value weights $\boldsymbol{w} \in \mathbb{R}^{d'}$ and policy parameter $\boldsymbol{\theta} \in \mathbb{R}^{d''}$ (e.g., to $\mathbf{0}$)
Algorithm parameters: $\alpha^w > 0$, $\alpha^\theta > 0$, $\alpha^{\bar{R}} > 0$
Initialize $S \in S$ (e.g. to s_0)
Initialize $\Psi \in \mathbb{R}^d$ (e.g., to ψ_0 = neutral emotion)
Initialize $R^{(T)}, V^{(T)}$ (as empty lists)
loop forever (for each time-step)
    $X = (S, \Psi)$ ▷ Extended state
    $A \sim \pi(\cdot \mid X, \boldsymbol{\theta})$ ▷ Choose an action
    Take action A, observe S', R ▷ Observe new state and reward
    $\delta \leftarrow R - \bar{R} + \widehat{v}(S', \boldsymbol{w}) - \widehat{v}(S, \boldsymbol{w})$ ▷ Temporal difference error
    $\bar{R} \leftarrow \bar{R} + \alpha^{\bar{R}}\delta$ ▷ Update average reward
    $\boldsymbol{w} \leftarrow \boldsymbol{w} + \alpha^w \delta \nabla \widehat{v}(S, \boldsymbol{w})$ ▷ Adjust $\widehat{v}$
    $\boldsymbol{\theta} \leftarrow \boldsymbol{\theta} + \alpha^\theta \delta \nabla \ln \pi(A \mid S, \boldsymbol{\theta})$ ▷ Adjust π
    $S \leftarrow S'$ ▷ Update state
    $v \leftarrow \widehat{v}(S', \boldsymbol{w})$ ▷ Evaluate new state
    Store R, V in lists $R^{(T)}$, $V^{(T)}$
    if time-step $\geq T$ **then**
        $\Psi = e(R^{(T)}, V^{(T)})$ ▷ Generate emotion
    end if
end loop

Training of an emotional agent (Order IV - World-knowledgeability)

The most basic emotional agent of Order IV would count with an additional learned world model m, anticipating next states from the current state and the action then taken. The emotional encoder would thus observe a third value, measuring the accuracy of the latest predictions, as suggested in 1, 'Emotional orders'.

We provide Algorithm 3, a basic pseudocode where:

$S' = m(s, a) \in \mathcal{S}$ is the external state predicted while being in state $s \in \mathcal{S}$ and taking action $a \in \mathcal{A}$;

diff $(s, s') \in \mathbb{R}$ is a measure of the difference between two external states s and $s' \in \mathcal{S}$ (for example, Euclidean);

$D^{(T)} = [D_{t-T+1}, \ldots, D_t] \in \mathbb{R}^T$ is the list of latest T values of *diff* () observed at time t;

$\Psi = e(R^{(T)}, V^{(T)}, D^{(T)}) \in \mathbb{R}^d$ is the emotion encoded by e from the latest T values of R, V and D at time t.

Algorithm 3 Training of an Order IV emotional actor-critic agent (continuing tasks)

Input: a differentiable policy parameterization $\pi(a \mid s, \boldsymbol{\theta})$

Input: a differentiable state-value function parameterization $\widehat{v}(s, \boldsymbol{w})$

Input: a trained emotional encoder $e(R^{()}, V^{()}, D^{()})$

Input: a trained world model $m(s, a)$

Input: a state-distance function $\mathit{diff}(s, s')$

Initialize $\bar{R} \in \mathbb{R}$ (e.g. to 0)

Initialize state-value weights $\boldsymbol{w} \in \mathbb{R}^{d'}$ and policy parameter $\boldsymbol{\theta} \in \mathbb{R}^{d''}$ (e.g., to $\mathbf{0}$)

Algorithm parameters: $\alpha^w > 0$, $\alpha^\theta > 0$, $\alpha^{\bar{R}} > 0$

Initialize $S \in S$

Initialize $\Psi \in \mathbb{R}^d$ (e.g., to ψ_0 = neutral emotion)

Initialize $R^{(T)}, V^{(T)}, D^{(T)}$ (as empty lists)

loop forever (for each time-step)

  $X = (S, \Psi)$ ▷ Extended state

  $A \sim \pi(\cdot \mid X, \boldsymbol{\theta})$ ▷ Choose an action

  $\widehat{S}' \leftarrow m(S, A)$ ▷ Predict next state

  Take action A, observe S', R ▷ Observe new state and reward

  $\delta \leftarrow R - \bar{R} + \widehat{v}(S', \boldsymbol{w}) - \widehat{v}(S, \boldsymbol{w})$ ▷ Temporal difference error

  $\bar{R} \leftarrow \bar{R} + \alpha^{\bar{R}}\delta$ ▷ Update average reward

  $\boldsymbol{w} \leftarrow \boldsymbol{w} + \alpha^w \delta \nabla \widehat{v}(S, \boldsymbol{w})$ ▷ Adjust $\widehat{v}$

  $\boldsymbol{\theta} \leftarrow \boldsymbol{\theta} + \alpha^\theta \delta \nabla \ln \pi(A \mid S, \boldsymbol{\theta})$ ▷ Adjust π

  $S \leftarrow S'$ ▷ Update state

  $V \leftarrow \widehat{v}(S', \boldsymbol{w})$ ▷ Evaluate new state

  $D \leftarrow \mathit{diff}(\widehat{S}', S')$ ▷ World-state prediction error

  Store R, V, D in lists, $R^{(T)}$, $V^{(T)}$, $D^{(T)}$

  if time-step $\geq T$ **then**

    $\Psi = e(R^{(T)}, V^{(T)}, D^{(T)})$ ▷ Generate emotion

  end if

end loop

References

[1] Sutton, R.S., Barto, A.G.: Reinforcement Learning: An Introduction - Second Edition. A Bradford Book, Cambridge, MA, USA (2018)

[2] Barto, A.G., Sutton, R.S., Anderson, C.W.: Neuronlike elements that can solve difficult learning control problems. IEEE Transactions on Systems, Man, and Cybernetics **13**(5), 835–846 (1983)

[3] Montague, P.R., Dayan, P., Sejnowski, T.J.: A framework for mesencephalic dopamine systems based on predictive hebbian learning. The Journal of Neuroscience **16**(5), 1936–1947 (1996)

[4] Levenson, R.W.: Human emotions: A functional view. In: Ekman, P., Davidson, R.J. (eds.) The Nature of Emotion: Fundamental Questions, pp. 123–126. Oxford University Press, New York, NY (1994)

[5] Ekman, P.: Emotions Revealed: Recognizing Faces and Feelings to Improve Communication and Emotional Life. Times Books, New York, NY (2003)

[6] Chentanez, N., Barto, A., Singh, S.: Intrinsically motivated reinforcement learning. In: Advances in Neural Information Processing Systems, vol. 17, pp. 1281–1288 (2004)

[7] Bengio, Y.: Learning deep architectures for AI. Foundations and Trends in Machine Learning **2**(1), 1–55 (2009)

[8] Lange, S., Riedmiller, M.: Deep auto-encoder neural networks in reinforcement learning. In: The 2010 International Joint Conference on Neural Networks (IJCNN), pp. 1–8 (2010). IEEE

[9] Kingma, D.P., Welling, M.: Auto-Encoding Variational Bayes. Preprint at https://doi.org/10.48550/

arXiv.1312.6114 (2013)

[10] Hochreiter, S., Schmidhuber, J.: Long short-term memory. Neural Computation **9**(8), 1735–1780 (1997)

[11] Cho, K., Merriënboer, B., Gulcehre, C., Bahdanau, D., Bougares, F., Schwenk, H., Bengio, Y.: Learning phrase representations using RNN encoder–decoder for statistical machine translation. In: Proceedings of the 2014 Conference on Empirical Methods in Natural Language Processing (EMNLP), pp. 1724–1734. Association for Computational Linguistics, Doha, Qatar (2014)

[12] Bahdanau, D., Cho, K., Bengio, Y.: Neural Machine Translation by Jointly Learning to Align and Translate. Preprint at https://arxiv.org/abs/1409.0473 (2014)

[13] Vaswani, A., Shazeer, N., Parmar, N., Uszkoreit, J., Jones, L., Gomez, A.N., Kaiser, Ł., Polosukhin, I.: Attention is all you need. Advances in Neural Information Processing Systems **30**, 5998–6008 (2017)

[14] Damasio, A.: The Strange Order of Things: Life, Feeling, and the Making of Cultures. Pantheon Books, New York, NY (2018)

[15] Damasio, A.: Feeling & Knowing: Making Minds Conscious. Pantheon Books, New York, NY (2021)

[16] Kahneman, D., Krueger, A.B.: Developments in the measurement of subjective well-being. Journal of Economic Perspectives **20**(1), 3–24 (2006)

[17] Kahneman, D.: Thinking, Fast and Slow. Farrar, Straus and Giroux, New York (2011)

[18] Fehr, L., Russell, J.: Concept of emotion viewed from a prototype perspective. Journal of Experimental Psychology: General **113**(3), 464–486 (1984)

[19] Kleinginna, P.R., Kleinginna, A.M.: A categorized list of emotion definitions, with suggestions for a consensual definition. Motivation and Emotion **5**(4), 345–379 (1981)

[20] Keltner, D., Lerner, J.S.: In: Gilbert, D.T., Fiske, S.T., Lindzey, G. (eds.) Emotion, pp. 317–352. John Wiley & Sons, Ltd, New York (2010)

[21] Schultz, W.: Neuronal reward and decision signals: From theories to data. Physiological Reviews **95**(3), 853–951 (2015)

[22] Russell, J.A., Mehrabian, A.: Evidence for a three-factor theory of emotions. J. Res. Personal. **11**, 273–294 (1977)

[23] Bradley, M.M., Lang, P.J.: Affective norms for english words (anew): Instruction manual and affective ratings. Technical Report C-1, Center for Research in Psychophysiology, University of Florida (1999)

[24] Redondo, J., Fraga, I., Padrón, I., Comesaña, M.: The spanish adaptation of anew (affective norms for english words). Behavior Research Methods **39**(3), 600–605 (2007)

[25] Landowska, A.: Towards new mappings between emotion representation models. Applied Sciences **8**(2), 274 (2018)

[26] Scott, G.G., Keitel, A., Becirspahic, M., *et al.*: The glasgow norms: Ratings of 5,500 words on nine scales. Behavior Research Methods **51**, 1258–1270 (2019)
